# p38α MAPK regulates proliferation and differentiation of osteoclast progenitors and bone remodeling in an aging-dependent manner

**DOI:** 10.1038/srep45964

**Published:** 2017-04-06

**Authors:** Qian Cong, Hao Jia, Ping Li, Shoutao Qiu, James Yeh, Yibin Wang, Zhen-Lin Zhang, Junping Ao, Baojie Li, Huijuan Liu

**Affiliations:** 1 Dept. of Osteoporosis and Bone Diseases, Shanghai Key Clinical Center for Metabolic Disease, Shanghai Jiao Tong University Affiliated Sixth People’s Hospital, Shanghai 200233, China; 2 Bio-X Institutes, Key Laboratory for the Genetics of Developmental and Neuropsychiatric Disorders, Ministry of Education, Shanghai Jiao Tong University, Shanghai 200240, China; 3 Department of Biochemistry and Molecular cellular Biology Shanghai Jiaotong University, School of Medicine, 280 Chongqing Rd, Shanghai, 200025, China; 4 Division of Molecular Medicine, Departments of Anesthesiology, Medicine and Physiology, Molecular Biology Institute, Cardiovascular Research Laboratories, David Geffen School of Medicine, Los Angeles, CA90095, USA; 5 State Key Laboratory of Oncogenes and Related Genes, Shanghai Cancer Institute, Renji Hospital, Shanghai Jiao Tong University, School of Medicine, Shanghai, China

## Abstract

Bone mass is determined by the balance between bone formation, carried out by mesenchymal stem cell-derived osteoblasts, and bone resorption, carried out by monocyte-derived osteoclasts. Here we investigated the potential roles of p38 MAPKs, which are activated by growth factors and cytokines including RANKL and BMPs, in osteoclastogenesis and bone resorption by ablating p38α MAPK in LysM+monocytes. p38α deficiency promoted monocyte proliferation but regulated monocyte osteoclastic differentiation in a cell-density dependent manner, with proliferating p38α^−/−^ cultures showing increased differentiation. While young mutant mice showed minor increase in bone mass, 6-month-old mutant mice developed osteoporosis, associated with an increase in osteoclastogenesis and bone resorption and an increase in the pool of monocytes. Moreover, monocyte-specific p38α ablation resulted in a decrease in bone formation and the number of bone marrow mesenchymal stem/stromal cells, likely due to decreased expression of PDGF-AA and BMP2. The expression of PDGF-AA and BMP2 was positively regulated by the p38 MAPK-Creb axis in osteoclasts, with the promoters of PDGF-AA and BMP2 having Creb binding sites. These findings uncovered the molecular mechanisms by which p38α MAPK regulates osteoclastogenesis and coordinates osteoclastogenesis and osteoblastogenesis.

Osteoporosis is a common disorder caused by enhanced bone resorption, decreased bone formation, or both^1^,^2^,^3^. The bone undergoes constant remodeling, with old bone being resorbed by osteoclasts and new bone formed by osteoblasts^4^. Osteoblasts are derived from mesenchymal stem/stromal cells (MSCs), which are defined by lineage-specific transcription factors Runx2 and Osterix^5^,^6^,^7^, while osteoclasts are derived from the hematopoietic stem cell-monocyte lineage, which requires transcription factors such as PU.1, NF-κB, c-Fos, and NFATc1^8^. How differentiation of osteoclasts and osteoblasts from their respective progenitors is regulated remains not fully understood.

Bone formation and resorption are coupled via complex mechanisms. MSCs and osteoblasts have been shown to synthesize and secrete M-CSF, RANKL, and OPG to regulate osteoclast differentiation from monocytes, with the ratio of RANKL to OPG determining the yield of osteoclasts^9^,^10^,^11^. Moreover, some of the osteogenic regulators, e.g., TGFβ1 and IGF1, are synthesized by osteoclasts and/or released from bone matrix during bone resorption^12^,^13^,^14^,^15^. While how MSCs/osteoblasts regulate osteoclastogenesis has been extensively studied, much less is known about how monocytes regulate osteoblastogenesis.

p38 MAPKs are activated by many cytokines and growth factors, some of which are important regulators of bone modeling and remodeling, e.g., RANKL,TGFβ1, BMPs, and TNFα^16^,^17^,^18^,^19^. p38 MAPKs have been shown to play important roles in MSC osteoblast commitment and maturation as well as proliferation^20^,^21^,^22^. In addition, p38 MAPKs can be activated by estrogen in MSCs, which controls the expression of OPG to regulate osteoclastogenesis and bone resorption^23^. However, the physiological roles of p38 MAPKs in osteoclastogenesis and bone resorption are less understood. RANKL activates p38 MAPKs via Rack1, Tak1, and MKK3/6^24^,^25^. Tak1 and MKK3/6 have been shown to play positive roles in osteoclast proliferation, differentiation, and/or survival^26^,^27^,^28^,^29^. Inhibitor or siRNA-based studies suggest that p38 MAPKs also play a positive role in RANKL-induced osteoclast differentiation^30^,^31^,^32^. Using a transgenic mouse line that overexpresses TNFα, several studies have shown that p38 MAPKs are involved in inflammation-induced bone loss, by controlling IL-1 expression and osteoclast differentiation^32^,^33^,^34^,^35^. Yet there is still a lack of genetic evidence whether p38 MAPKs play an important role in osteoclastogenesis from its progenitors under physiological conditions.

Here we ablated p38α, the essential p38 isoform, in monocytes using a LysM-Cre mouse line^17^,^36^,^37^, and found that p38α ablation increased monocyte proliferation but could stimulate or inhibit monocyte osteoclastic differentiation depending on cell density and the proliferation status. Phenotypically, the mutant mice showed a minor increase in bone mass at 2.5 month of age but develop osteoporotic phenotypes at 6 month of age. The modest increase in bone mass in young mice is associated with a decrease in the number of osteoclasts and bone resorption rate, which can only be explained by compromised osteoclast differentiation. The decrease in bone mass in 6-month-old mice is associated with an increase in the number of osteoclasts and bone resorption rate, due to an expansion of the monocyte pool and likely enhanced osteoclast differentiation. Furthermore, p38α deficiency in LysM+ progenitors/osteoclasts suppressed the synthesis of PDGF-AA and BMP2, leading to a defect in MSC proliferation and differentiation and decreased bone formation. Mechanistic studies show that p38α regulates PDGF-AA and BMP2 expression in osteoclasts via Creb, which have multiple binding sites in the promoters of these two genes. Our findings collectively suggest that p38α plays important roles in osteoclastogenesis by coordinating monocyte proliferation and differentiation and in coupling osteoclastogenesis and osteoblastogenesis.

## Results

### p38α deficiency promoted monocyte proliferation

**Figure 1:**
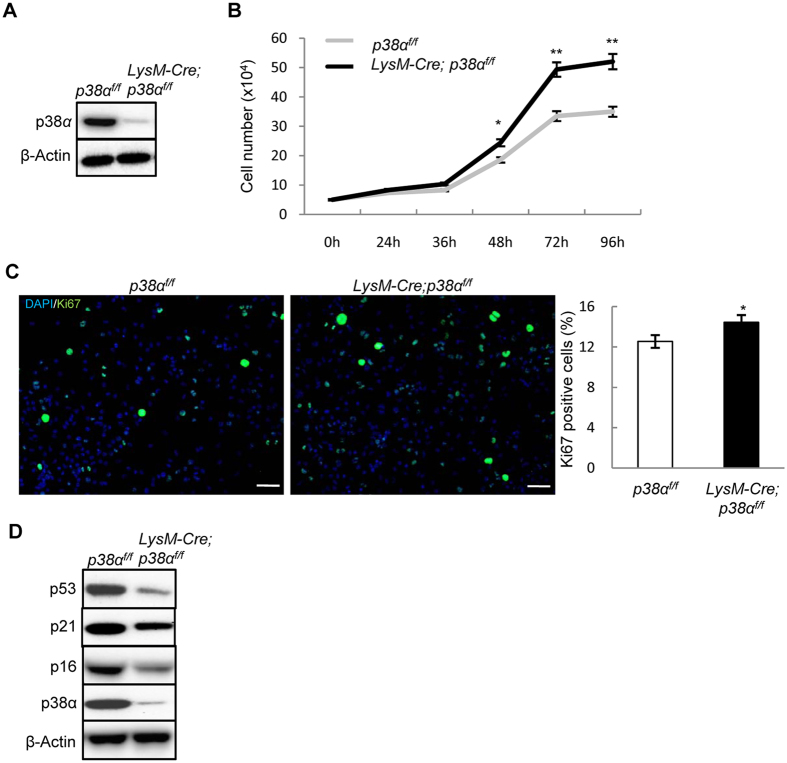
p38α deficiency promoted monocyte proliferation and osteoclast differentiation. (**A**) Western blot results showed that p38α was deleted in the monocyte cultures of the LysM-Cre; p38α^f/f^ mice. Bone marrow monocytes were isolated from mutant and control mice, cultured in the presence of M-CSF and RANKL for 2 days, and then used for western blot analysis. (**B**) p38α deficient monocytes showed increased proliferation. Bone marrow monocytes were isolated from the mutant and control mice, plated at the same number, cultured in the presence of M-CSF/RANKL, and counted at different time points. N = 3. (**C**) p38α deficiency increased cell proliferation in monocyte cultures. WT and p38α deficient monocytes were induced to differentiate by M-CSF/RANKL. At day 2, these cells were immunostained for Ki67 to detect S phase cells (left panel). The ratios of Ki67 positive cells to DAPI-stained cells were presented (right panel). Scale bar, 50 μm. N = 3. (**D**) Western blot analysis of extracts of cells described in Fig. 1A revealed that p38α deficient monocyte cultures showed a decrease in the levels of anti-proliferation proteins p53, p21, and p16. For all results in Fig. 1, P-values are based on Student’s t-test. *p < 0.05, **p < 0.01 when the value of mutant mice or cells was compared to that of control mice or cells.

To test whether p38α plays a role in osteoclastogenesis *in vivo*, we ablated p38α with a LysM-Cre mouse line, which has been used to study monocytes, the osteoclast progenitors^38^,^39^. Western blot analysis revealed that the protein levels of p38α in monocyte cultures derived from LysM-Cre; p38α^f/f^ mice were about 8% of those of wild type cells (Fig. 1A), suggesting that more than 90% of the cells had p38α deletion. p38α deficiency increased the proliferation of *ex vivo* monocytes, judged by an increase in the number of total cells and Ki67 positive S phase cells in cultures (Fig. 1B and C), which were associated with a decrease in p53, p21, and p16 (Fig. 1D), negative regulators of cell proliferation that are regulated by p38 MAPKs^36^. On the other hand, the number of apoptotic cells in the cultures was not significantly altered by p38α deficiency (Supplementary Fig. S1A).

### p38α could positively or negatively regulate osteoclast differentiation

**Figure 2:**
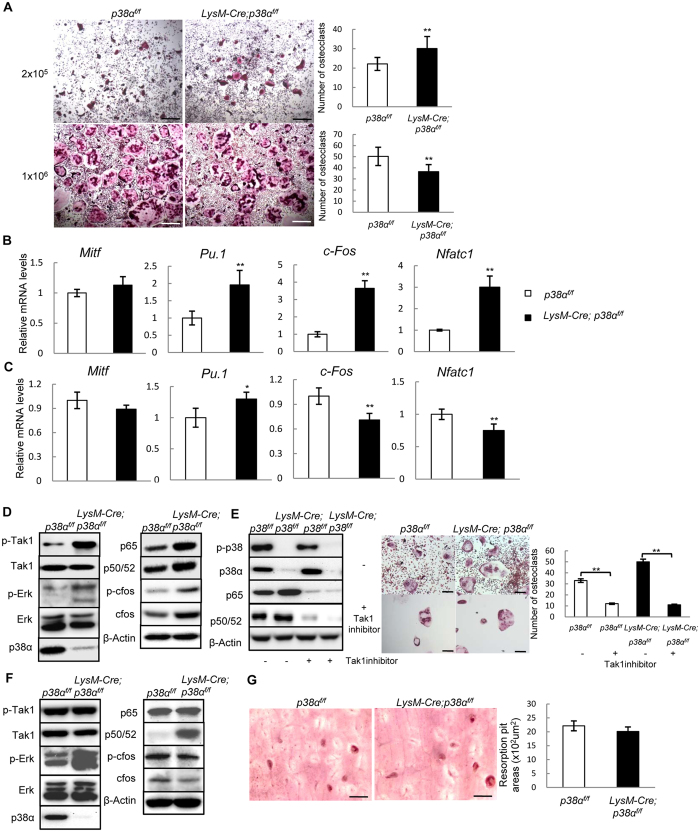
p38α regulated osteoclast differentiation in a cell density-dependent manner. (**A**) TRAP staining showed that p38α deficiency promoted osteoclast differentiation in low cell density culture but slightly inhibited osteoclast differentiation in high cell density cultures. Monocytes were isolated from LysM-Cre; p38α^f/f^ and control mice, plated at different densities, and cultured in the presence of RANKL and M-CSF. After 7 days, the cultures were stained for TRAP (left panels). Right panels: quantitation data. Scale bar, 200 μm. N = 3. (**B**) Quantitative PCR results showed that p38α deficiency promoted the expression of genes required for osteoclast differentiation at low cell densities. Monocytes described in Fig. 2A (low cell density) were collected and used to isolate total RNA, which was used to run quantitative PCR assays. N = 3. (**C**) Quantitative PCR results showed that p38α deficiency slightly inhibited the expression of genes required for osteoclast differentiation at high cell densities. (**D**) Western blot results showed that p38α deficient monocytes cultures at low densities exhibited an increase in activation of Tak1 and the protein levels of NF-κB isoforms p50/52 and p65. Scale bar, 100 μm. (**E**) Inhibition of Tak1 down-regulated NF-κB levels and suppressed osteoclast differentiation. Left panel: western blot showed that Tak1 inhibitor down-regulated NF-κB levels. Middle panel: Tak1 inhibitor suppressed osteoclast differentiation. Right panel: Quantitation data. N = 3. (**F**) Western blot results revealed that p38α deficient monocyte cultures at high densities showed a decrease in c-Fos protein levels. (**G**) p38α deficiency did not affect the resorbing activity of osteoclasts on dentine slices. WT and p38 deficient monocytes were induced to differentiate into osteoclasts by M-CSF and RANKL for 2 days and then counted and the same numbers of cells were plated onto dentine slices. After 7 days, the dentine slices were sonicated and stained with Gill’s hematoxylin. Right panel: quantitation data. Scale bar, 200 μm. N = 3. For all results in Fig. 2, P-values are based on Student’s t-test. *p < 0.05, **p < 0.01 when the value of mutant mice or cells was compared to that of control mice or cells, or the drugs-treated group compared to control group.

We then compared the differentiation potential of p38α deficient monocytes isolated from 2.5-month-old mice. We found that in response to M-CSF and RANKL, p38α deficiency either stimulated or impeded osteoclast differentiation depending on the cell densities. When plated at a low density, p38α deficient monocyte cultures showed an increase in the number of TRAP positive osteoclasts (Fig. 2A), and an increase in the mRNA levels of PU.1, c-Fos, and NFATc1, transcription factors required for osteoclastogenesis (Fig. 2B). However, when plated at a high density, p38α deficient monocyte cultures showed a modestly decreased number of TRAP positive osteoclasts and a decrease in the mRNA levels of c-Fos and NFATc1 (Fig. 2A and C). Note that at high cell densities, osteoclasts appeared to be smaller than the ones in low-density cultures, suggesting that the limit of space may have interfered with osteoclast growth and/or differentiation, which warrants further investigation. Moreover, since cells at high densities do not usually have space to proliferate, these results also suggest that p38α’s roles in regulating early osteoclast differentiation may be linked to its effects on cell proliferation.

We found that p38α deficient monocyte cultures, at a low density, showed an increase in the protein levels of p50/52 and p65 isoforms of NF-κB, potent stimulators of osteoclast differentiation, and enhanced activation of Tak1, an upstream activator of NF-κB (Fig. 2D), enhanced Erk activation, which can be activated by Tak1 (Fig. 2D). Previous studies have shown that p38 MAPKs phosphorylate Tab1/2 to inhibit Tak1 activation^36^,^40^, and Tak1 plays a positive role in osteoclast proliferation, differentiation, and survival^26^,^27^,^28^,^29^. It is likely that enhanced Tak1 activation may mediate the effects of p38α deficiency on osteoclastogenesis. Indeed, we found that inhibition of Tak1 with 5Z-7-Oxozeaenol down-regulated NF-κB molecules and impeded osteoclast differentiation of p38α deficient and WT monocytes (Fig. 2E). These results suggest that p38α deficiency promoted osteoclastogenesis via hyperactivating Tak1 and NF-κB at low cell densities.

However, at a high cell density, p38α deficiency did not affect Tak1 activation or the p65 isoform of NF-κB, although it led to a decrease in the protein levels of c-Fos and phospho-c-Fos and an increase in p50/52 NF-κB (Fig. 2F). These results suggest that Tak1 and NF-κB may not be involved in regulating osteoclast differentiation in high density cultures. Instead, down-regulation of c-Fos may contribute to the modest suppression of osteoclast differentiation caused by p38α deficiency. In addition, we found that when the same number of differentiated osteoclasts were plated on dentine slices, p38α deficiency did not show an effect on *in vitro* osteoclast bone resorption activity (Fig. 2G), consistent with previous reported results^30^. Moreover, although p38 MAPK-mediated expression of DC-Stamp has been shown to mediate vitamin E-induced osteoclast fusion^41^, we did not find an alteration in DC-Stamp expression and osteoclast fusion in the absence of p38α (Fig. 2A and Supplementary Fig. S1B).

The above findings suggest that effect of p38α deficiency on osteoclast differentiation may be related to its effects on cell proliferation. Moreover, p38α deficiency only caused a modest effect on osteoclast differentiation in high cell density cultures, this is inconsistent with previous studies reporting that small molecule compound p38 MAPK inhibitors such as SB203580 greatly inhibited osteoclast differentiation^30^,^42^. We repeated the inhibitor experiment in high density monocyte cultures and found that the commonly used p38α/β inhibitor SB203580 inhibited differentiation of not only WT but also p38α deficient osteoclasts, to similar extents (Supplementary Fig. S2). Since SB203580 inhibits both p38α and p38β, we tested whether p38β played a role in osteoclast differentiation. p38β was expressed in osteoclasts, yet knockdown of p38β did not significantly affect osteoclast differentiation in WT or p38α deficient monocytes (Supplementary Fig. S3A and S3B). Although it is possible that this result is caused by SB203580 targeting the leftover p38α MAPKs in LysM-Cre; p38α^f/f^ monocytes, our observation that more than 90% of the monocytes had p38α deletion (Fig. 1A) argues against it. Instead, SB203580 may target other kinases besides p38α/β MAPKs and that the roles of p38α/β in osteoclast differentiation are not as great as previously thought.

### LysM-Cre; p38α^f/f^ mice developed osteoporotic phenotypes at 6 month of age

**Figure 3:**
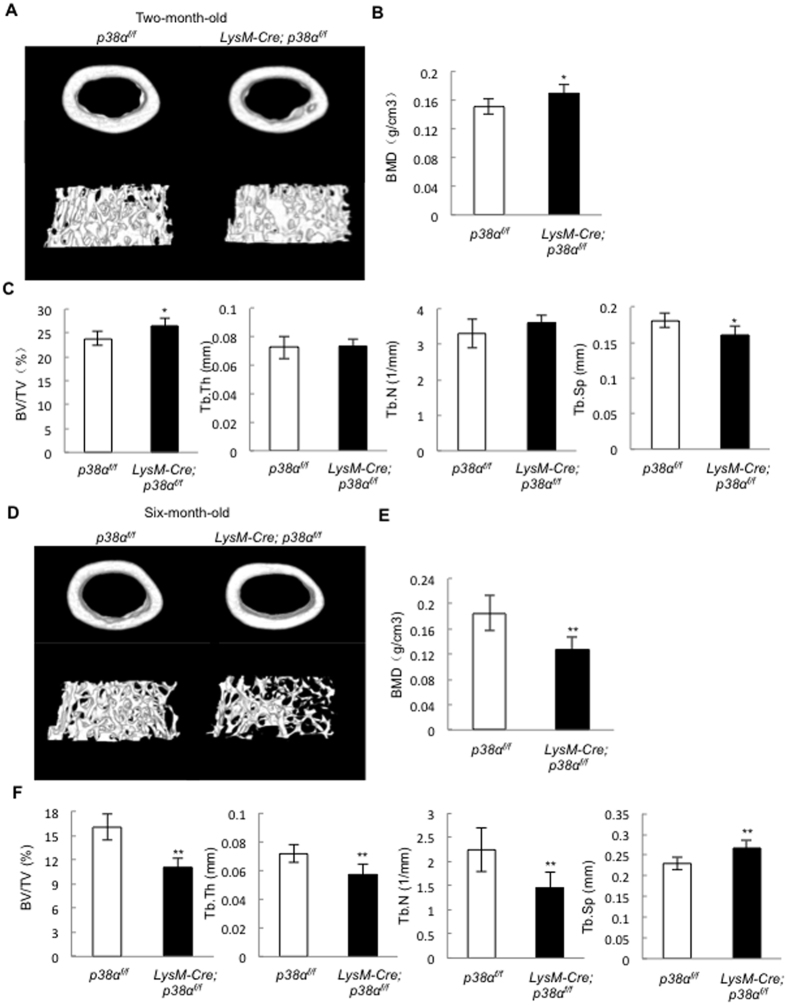
Micro-CT results revealed that LysM-Cre; p38α^f/f^ mice developed osteoporotic phenotypes at 6 month of age. (**A**) A representative μCT image of the femur bones of 2.5 month-old LysM-Cre; p38α^f/f^ and control mice. (**B**) Two and half-month-old LysM-Cre; p38α^f/f^ mice, compared to control mice, showed a minor increase in bone mineral density in the femur trabecular bones. N = 8. (**C**) Two and half-month-old LysM-Cre; p38α^f/f^ mice only showed a minor increase in BV/TV and a decrease in trabecular separation, but not a change in trabecular number or thickness compared to control mice. N = 8. (**D**) A representative μCT image of the femur bones of 6 month-old LysM-Cre; p38α^f/f^ and control mice. (**E**) Bone mineral density of the femur trabecular bones of 6 month-old LysM-Cre; p38α^f/f^ and control mice. N = 8. (**F**) Six-month-old LysM-Cre; p38α^f/f^ mice showed a decrease in BV/TV, trabecular number, trabecular thickness, and an increase in trabecular separation compared to control mice. N = 8. For all results in Fig. 3, P-values are based on Student’s t-test. *p < 0.05, **p < 0.01 when the value of mutant mice was compared to that of control mice.

To test whether p38α ablation affected osteoclastogenesis and bone remodeling *in vivo*, we analyzed the bone parameters of mutant and age-matched control mice. The mutant mice were born at expected Mendelian frequency, with normal body size and limb length (Supplementary Fig. S4A and S4B). Micro-CT analysis revealed that at 2.5 month of age, the mutant mice showed a minor increase (less than 10%) in BMD and bone volume, without affecting trabecular bone number and thickness or cortical bone BMD and bone volume (Fig. 3A–C). Bone histomorphometry analysis confirmed that the mutant mice showed a minor increase in bone volume without affecting trabecular bone number, thickness, or separation (Supplementary Fig. S5A and S5B).

However, micro-CT analysis revealed that 6-month-old mutant mice showed osteoporotic phenotypes, with a decrease (about 30%) in BMD, bone volume, trabecular bone number, and trabecular bone thickness, as well as an increase in trabecular separation (Fig. 3D–F). The bone histomorphometric results confirmed that the 6-month-old mutant mice showed a decrease in bone volume, trabecular bone number, trabecular bone thickness, and an increase in trabecular separation (Supplementary Fig. S5C and S5D). These results suggest that p38α expressed in monocytes and osteoclasts regulate bone remodeling in an age-dependent manner.

### Bone resorption in LysM-Cre; p38α^f/f^ mice was increased at 6 month of age

**Figure 4:**
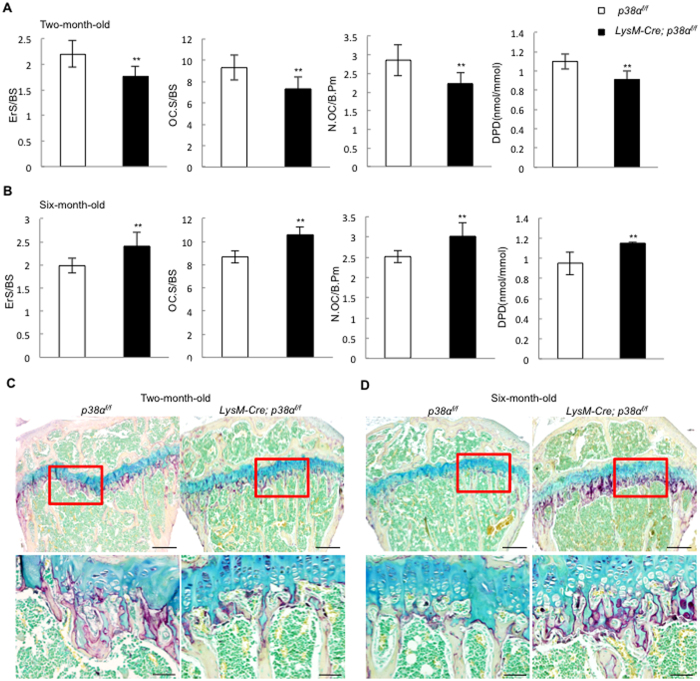
LysM-Cre; p38α^f/f^ mice showed distinct alterations in bone resorption at 2.5 and 6 month of age. (**A**) Two and half-month-old LysM-Cre; p38α^f/f^ mice showed a slight decrease in erosion surface, osteoclast surface, osteoclast number, and urine DPD levels compared to control mice. N = 8. (**B**) Six-month-old LysM-Cre; p38α^f/f^ mice showed an increase in erosion surface, osteoclast surface, osteoclast number, and urine DPD levels compared to control mice. N = 8. (**C**) *In vivo* TRAP staining showed that the femur bones of 2.5-month-old LysM-Cre; p38α^f/f^ mice exhibited a minor decrease in osteoclasts compared to control mice. Scale bar, 200 μm. N = 3. (**D**) *In vivo* TRAP staining showed that the femur bones of 6-month-old LysM-Cre; p38α^f/f^ mice exhibited an increase in osteoclasts compared to control mice. Scale bar, 50 μm. N = 3. For all results in Fig. 4, P-values are based on Student’s t-test. **p < 0.01 when the value of mutant mice or cells was compared to that of control mice.

Histomorphometry analysis showed that the modest increase in bone mass in 2.5-month-old mice was associated with a decrease in bone resorption (Fig. 4A). The bone erosion surfaces and the number of osteoclasts per bone surface were decreased (Fig. 4A). In addition, the levels of urine DPD, an *in vivo* marker of bone resorption, was also decreased (Fig. 4A). The decrease in the number of osteoclasts could only be explained by compromised differentiation of osteoclasts, as p38α deficiency promoted monocyte proliferation.

On the other hand, the osteoporotic phenotypes of 6-month-old LysM-Cre; p38α^f/f^ mice were associated with an increase in bone resorption (Fig. 4B). The erosion surface, the number of osteoclasts per bone surface, and the levels of urine deoxypyridinoline (DPD) were all increased compared to the age-matched control mice (Fig. 4B). This could be explained by increased monocyte proliferation, increased osteoclast differentiation, or both. To validate this finding, we stained tibia bones of 2.5 and 6-month-old LysM-Cre; p38α^f/f^ and WT mice for TRAP, and found that TRAP staining was slightly decreased in 2.5 month of age (Fig. 4C), but greatly increased at 6 month of age, in the mutant mice (Fig. 4D). These results suggest that ablation of p38α affects osteoclastogenesis and bone resorption in an age-dependent manner.

### LysM-Cre; p38α^f/f^ mice showed an increased monocyte pool at 6 month of age

How does p38α deficiency dissimilarly affect osteoclastogenesis at different ages? To test whether age difference altered osteoclast differentiation potential, we isolated primary monocytes from 6-month-old LysM-Cre; p38α^f/f^ and control mice. These p38α deficient monocytes, like those isolated from 2.5-month-old mice (Fig. 2), showed increased proliferation *in vitro* compared to control monocyte cultures (Supplementary Fig. S6A). At low densities, p38α deficiency promoted osteoclast differentiation, whereas at high densities, p38α deficiency slightly inhibited osteoclast differentiation (Supplementary Fig. S6B). In addition, p38α deficiency did not affect bone resorption activity of osteoclasts on dentine slices (Supplementary Fig. S6C). Thus, monocytes isolated from 2.5- or 6-month-old mice displayed similar proliferation and differentiation behaviors *in vitro*.

**Figure 5:**
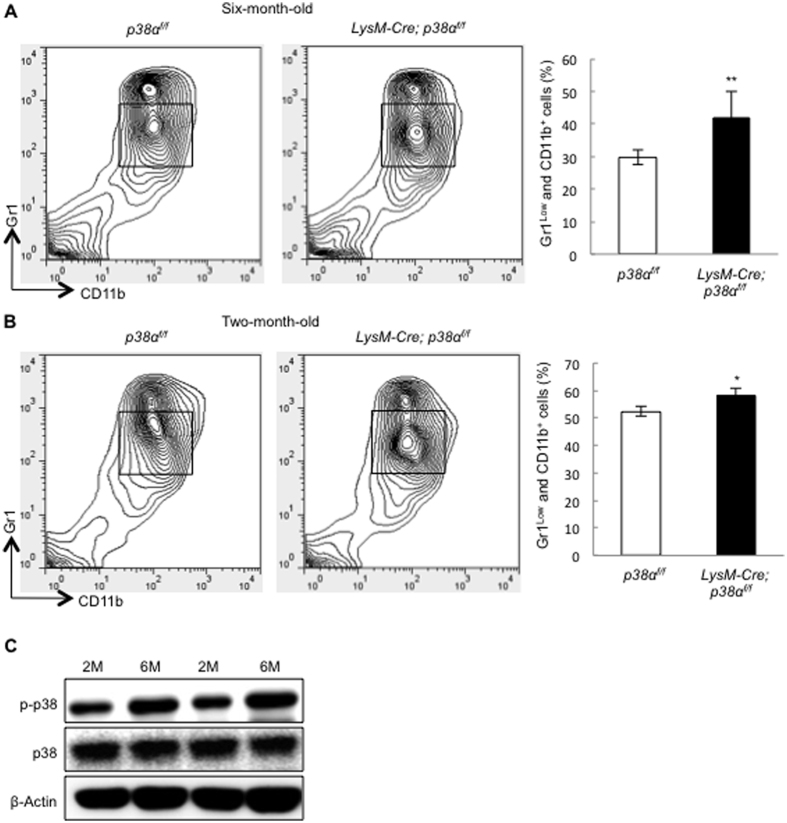
LysM-Cre; p38α^f/f^ mice showed an increase in the size of monocyte pool. (**A**) The number of the bone marrow Gr1^low^CD11b^+^ monocytes was increased in 6 month-old LysM-Cre; p38α^f/f^ mice compared to control mice. N = 3. (**B**) The number of the bone marrow Gr1^low^CD11b^+^ monocytes was increased in 2.5 month-old LysM-Cre; p38α^f/f^ mice compared to control mice, to a lesser extent than that of 6-month-oldmice. N = 3. (**C**) Western blot showed that p38 MAPK activation was enhanced in the bone marrow monocytes isolated from 6-month-old normal mice compared to that of 2.5-month-old mice. BM cells were flushed out of the bones and purified with the Percoll method at 4 ^o^C, which were directly used for western blot analysis. Results from two pairs of mice were shown. For all results in Fig. 5, P-values are based on Student’s t-test. *p < 0.05, **p < 0.01 when the value of mutant mice was compared to that of control mice.

Since p38α deficiency promotes monocyte proliferation, we compared the numbers of bone marrow monocytes between LysM-Cre; p38α^f/f^ and age-matched control mice. Bone marrow monocytes can be labeled by Gr1^low^CD11b^+^ 43. Flow cytometry analysis revealed that the percentage of monocytes in bone marrow was increased in 6-month-old LysM-Cre; p38α^f/f^ mice compared to the age-matched control mice (Fig. 5A). The percentage of bone marrow monocytes were also increased in 2.5-month-old LysM-Cre; p38α^f/f^ mice, yet to a much lesser extent than 6 month-old mice (Fig. 5B).

The greater increase in the number of bone marrow monocytes in 6-month-old mouse may reflect accumulation of overproliferating p38α deficient monocytes over time. Interestingly, comparison of p38 MAPK activation in monocytes isolated from 2.5 and 6-month-old normal mice revealed that p38 MAPK activation was enhanced in monocytes of the older mice (Fig. 5C). Since monocytes isolated from 2.5- or 6-month-old mice showed similar behaviors *in vitro* (Supplementary Fig. S6), we suspect that the difference might be attributable to environmental changes in older mice. An increase in p38 MAPK activation in older mice has been reported in a few other tissues, which may reflect the accumulation of stress^44^,^45^. Enhanced p38 MAPK activation may restrict monocyte proliferation in older mice to a greater extent than that in young mice; p38α deficiency may relieve this restriction and lead to greater expansion of monocytes in older mice than in young mice. The growth difference caused by p38α ablation at different ages may contribute to the difference in osteoclast differentiation (Fig. 2).

### LysM-Cre; p38α^f/f^ mice showed a decrease in bone formation

**Figure 6:**
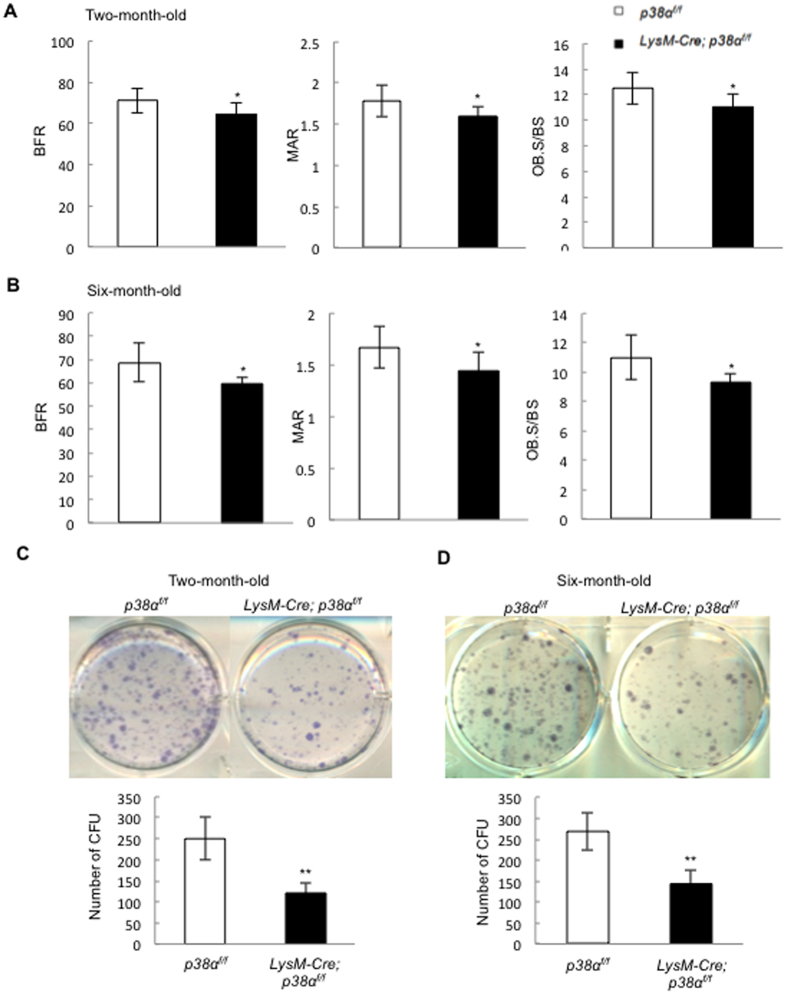
LysM-Cre; p38α^f/f^ mice showed a decrease in bone formation at 2.5 or 6 month of age. (**A**) Two and half-month-old LysM-Cre; p38α^f/f^ mice showed a decrease in BFR, MAR, and the number of osteoblasts per bone surface compared to control mice. N = 8. (**B**) Six-month-old LysM-Cre; p38α^f/f^ mice showed a decrease in BFR, MAR, and the number of osteoblasts per bone surface compared to control mice. N = 8. (**C**) Two and half-month-old LysM-Cre; p38α^f/f^ mice showed a decrease in the number of bone marrow colony forming units compared to control mice. Upper panel: ALP staining. Bottom panel: quantitation data. N = 3. (**D**) Six-month-old LysM-Cre; p38α^f/f^ mice showed a decrease in the number of bone marrow colony forming units compared to control mice. Upper panel: ALP staining. Bottom panel: quantitation data. N = 3. For all results in Fig. 6, P-values are based on Student’s t-test. *p < 0.05, **p < 0.01 when the value of mutant mice or cells was compared to that of control mice or cells.

Histomorphometry analysis also revealed that in 2.5- or 6-month-old mutant mice, the bone formation rate, mineral apposition rate, and the number of osteoblasts were all decreased compared to age-matched control mice (Fig. 6A and B). Since LysM does not label MSCs or osteoblasts, these results suggest that p38α deficiency may indirectly affect osteoblast proliferation and differentiation, for instance, by affecting the expression and secretion of coupling factors. We also compared the number of bone marrow ALP positive CFUs in LysM-Cre; p38α^f/f^ and normal control mice, which is an indication of the number of MSCs, and found that p38α ablation in monocytes and osteoclasts led to a significant decrease in the number of MSCs in 2.5 or 6-month-old mice (Fig. 6C and D). These results suggest that p38α may regulate osteoclast synthesis and secretion of growth factors to regulate MSC proliferation and/or differentiation.

### p38α deficient monocytes show reduced capacity in supporting MSC differentiation

**Figure 7:**
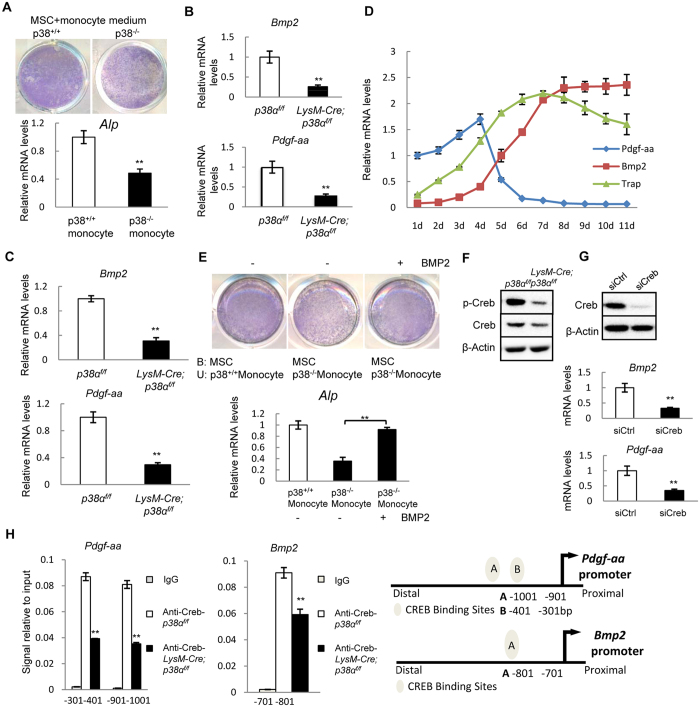
Ablation of p38α in monocytes promoted osteoblastogenesis by promoting BMP2/PDGF-AA synthesis via Creb. (**A**) p38α deficient monocyte culture medium showed a reduced activity in supporting osteogenic differentiation of normal MSC cells. Normal MSCs were plated and 24 hrs later, culture medium of WT or p38α deficient monocytes (in the absence of RANKL/M-CSF) was added to the cultures. Four days later, the plates were stained for ALP. Bottom panel: quantitation data of ALP. N = 3. (**B**) Quantitative PCR results showed that p38α deficient monocytes exhibited a decrease in the mRNA levels of BMP2 and PDGF-AA compared to control cells. N = 3. (**C**) Quantitative PCR assays revealed that the bone extracts of LysM-Cre; p38α^f/f^ mice showed a decrease in the mRNA levels of PDGF-AA and BMP2 compared to control mice. N = 3. (**D**) Expression of PDGF-AA, BMP2 and TRAP during osteoclast differentiation. The WT monocytes were induced to differentiate by M-CSF/RANKL. The cells were harvested at different times of induction and total RNA was isolated from these cells, which was used to perform quantitative PCR assays. The basal levels of PDGF-AA mRNA were set at 1.0 and the relative amounts of BMP2 and TRAP were normalized to that of PDGF-AA. (**E**) Addition of BMP2 to the co-culture system of WT MSCs and p38α deficient monocytes enhanced osteoblast differentiation, justified by ALP staining. Bottom Panel: ALP quantitation data. N = 3. (**F**) Western blot results show that p38α deficient monocytes displayed a decrease in the levels of p-Creb compared to WT cells. (**G**) Knockdown of Creb with siRNA led to down-regulation of PDGF-AA and BMP2 expression in normal monocytes. Upper panel: western blot showing the knockdown of Creb. Bottom panel: quantitation data. N = 3. (**H**) Chromatin-IP experiments showed that PDGF-AA promoter had two Creb binding sites and BMP2 promoter had one Creb binding sites. Right panel: diagrams showing the binding sites of Creb in the promoters of *PDGF-AA* and *BMP2* genes. N = 3. For all results in Fig. 7, P-values are based on Student’s t-test. **p < 0.01 when the mutant mice were compared to control mice, or Creb knockdown cells were compared to the control cells.

We then carried out co-culture experiments of MSCs and monocytes to test whether ablation of p38α in monocytes had an effect on MSC differentiation. WT MSCs were plated first and 24 hrs later, monocytes were plated on top of the MSC layer. After 4 days, the suspension cells were washed off and the cell culture plates were stained for ALP, a marker of osteoblast differentiation. We found that p38α deficient monocytes and osteoclasts showed a reduced capacity in supporting MSC osteogenic differentiation, in the presence or absence of RANKL/M-CSF (Supplementary Fig. S7A and S7B). Moreover, the culture medium of p38α deficient monocytes also showed a lower capacity in supporting MSC osteogenic differentiation than the medium of WT osteoclast cultures (Fig. 7A). These results suggest that p38α deficient osteoclasts may secrete a reduced amount of a pro-osteogenic differentiation factor.

### p38α regulated BMP2/PDGF-AA expression via Creb in monocytes and osteoclasts

To identify the growth factors or cytokines secreted by monocytes and/or osteoclasts, we tested whether p38α deficiency altered the expression of the cytokines and growth factors that have been reported to couple osteoclastogenesis and osteoblastogenesis including Ephrin B2, Cthrc1, LIF, IL6, IL11, Wnt10B, TGFβ1, IGF1, PDGF-AA, PDGF-BB, BMP2, BMP6, and BMP4^12^. Quantitative PCR analysis showed that p38α deficient monocytes expressed greatly reduced levels of BMP2 and PDGF-AA, modestly reduced TGFβ1 and IGF1, but not other molecules tested (Fig. 7B and Supplementary Fig. S8A). Moreover, bone extracts also showed a decrease in BMP2 and PDGF-AA mRNA levels when p38α is deleted in osteoclasts (Fig. 7C). In the course of osteoclast differentiation, PDGF-AA was slightly up-regulated before the expression of TRAP, whereas BMP2 was up-regulated after TRAP expression (Fig. 7D). Decreased PDGF-AA expression may contribute to the decrease in the number of MSCs in LysM-Cre; p38α^f/f^ mice. Moreover, addition of BMP2 rescued the decreased osteogenic differentiation of WT MSCs co-cultured with p38α deficient monocytes (Fig. 7E). On the other hand, TGFβ1 and IGF1 were expressed at low levels (required >40 PCR cycles to detect) in monocytes or osteoclasts and were not affected by osteoclast differentiation stages (Supplementary Fig. S8B and S8C).

To identify the transcription factors downstream of p38α that regulate the expression of PDGF-AA and BMP2, we knocked down each of the well-studied transcription factors in RAW264.3 cells and looked at their effect on the mRNA levels of PDGF-AA and BMP2 (data not shown). We found that Creb appeared to mediate the p38α’s effect on BMP2 and PDGF-AA expression. Western blot showed that Creb phosphorylation was reduced in p38α deficient monocytes and osteoclasts (Fig. 7F). Knockdown of Creb with siRNA in osteoclasts led to a decrease in the mRNA levels of PDGF-AA and BMP2 (Fig. 7G). ChIP assays showed that there were 2 Creb binding sites in PDGF-AA promoter and 1 binding site in BMP2 promoter (Fig. 7H). These results collectively suggest that osteoclasts utilized the p38α-Creb axis to regulate the expression of PDGF-AA and BMP2 to further influence MSC proliferation and differentiation.

## Discussion

p38 MAPKs can be activated by growth factors and cytokines, some of which, e.g., BMPs, TGFβ, and RANKL, play important roles in bone development and remodeling. In the MSC-osteoblast lineage, p38 MAPKs are found to promote MSC osteogenic commitment and osteoblast maturation by suppressing the NF-κB pathway and phosphorylating Runx2^20^,^37^,^46^. In addition, p38 MAPKs in Prx1-labeled MSCs appear to regulate the expression of OPG, which regulates bone mass accrual under physiological and pathological conditions^23^. However, the physiological functions of p38α in the monocyte-osteoclast lineage and bone resorption are not well understood. In this study, we ablated p38α in LysM-labeled monocytes and found that p38α inhibits monocyte proliferation and regulates osteoclastic differentiation in a cell density/proliferation-dependent manner. It also controls the expression of PDGF-AA and BMP2 in osteoclasts to affect osteoblastogenesis and bone formation. The coordinated actions of p38α in monocytes may be needed to maintain optimal bone mass. Disruption of p38α resulted in osteoporotic phenotypes at 6 month of age in mice while younger mice showed a minor increase in bone mass. This study thus provides genetic evidence that p38α plays complex roles in osteoclastogenesis, which are affected by the age of mice, and in coupling osteoclastogenesis and osteoblastogenesis.

Osteoclastogenesis is a multi-step process that includes expansion of the progenitors, commitment to osteoclasts, and cell fusion. p38α deficiency leads to enhanced monocyte proliferation, likely due to a reduction in the cyclin-dependent kinase inhibitors p16 and p21 as well as tumor suppressor p53, and an expansion of the bone marrow monocyte pool, especially in old mice. Moreover, we show for the first time that p38α affects *in vitro* osteoclast differentiation in a cell density dependent manner, which may be related to the proliferation status of the cells. In low cell density cultures, p38α deficiency promotes osteoclast differentiation, whereas in high cell density cultures, p38α deficiency mildly inhibits osteoclast differentiation. It has been reported that cell proliferation plays a positive role in promoting osteoclast differentiation^47^,^48^,^49^. In addition, p38α has been shown to play a role in sensing cell density and determines myoblast differentiation^50^,^51^. Moreover, low density cultures of p38α deficient monocytes showed an increase in Tak1 activation and NF-κB protein levels, whereas high density culture showed normal Tak1 activation. These results suggest that the density-dependent effect of p38α deficiency may be related to the proliferation potential that involves Tak1 activation, which warrants further investigation. Overall, our findings reveal much complex roles for p38α in osteoclastogenesis compared to the p38 MAPK inhibitor-based studies. Moreover, we also showed that p38β was expressed in the osteoclasts, yet it played a minor role in osteoclast differentiation.

Our genetic evidence suggests that p38α expressed in monocytes and osteoclasts coordinates osteoclastogenesis and osteoblastogenesis. When p38α is ablated in monocytes, bone formation is down-regulated regardless the age of the mice. p38α appears to regulate osteoclastogenesis-osteoblastogenesis coupling by controlling the expression of PDGF-AA and BMP2 in monocytes and osteoclasts via its downstream transcription factor Creb, which binds to the promoters of these two genes. Interestingly, monocytes, even in the absence of RANKL/M-CSF, synthesize PDGF-AA. Moreover, monocyte appears to express stage-specific coupling factors that have different effects on MSC proliferation and differentiation. The orderly expression of PDGF-AA and BMP2 by monocytes and osteoclasts may first facilitate MSC expansion and then osteogenic differentiation.

It has been long known that p38 MAPKs play an important role in promoting cell senescence and aging^52^,^53^,^54^. In some tissues, like bone marrow monocytes, an increase in the levels of p38 MAPK activation is associated with aging^44^,^45^,^55^. In this study, we found that p38 MAPKs are highly activated in monocytes of older mice compared to young mice, suggesting that p38α may display a stronger effect on osteoclastogenesis in old mice than in young mice and as such, p38 ablation may show more robust effects on monocyte proliferation and differentiation. This may be a reason why p38α deficient mice showed a great decrease in bone mass and developed osteoporosis at 6 month of age, attributable to an increase in osteoclastogenesis and bone resorption. These findings suggest that p38α plays a role in osteoclastogenesis in an age-dependent manner.

In summary, the present study demonstrates that p38α plays complex roles in monocyte proliferation and osteoclast differentiation in cell-autonomous manner, and in coupling osteoclastogenesis and osteoblastogenesis. Moreover, p38 MAPK activation in monocytes appears to be regulated in an age-dependent manner, which controls osteoclastogenesis by suppressing monocyte proliferation and differentiation and the size of the osteoclast progenitor pool. As such, p38α may mediate the effects of RANKL, BMPs, and other cytokines, on bone remodeling, and thus represent a drug target for osteoporosis therapy.

## Materials and Methods

### Mice and genotyping

The p38α^f/f^ mouse line was generated in Dr. Yibin Wang’s laboratory at UCLA. The LysM-Cre mouse line was purchased from The Jackson Laboratories. All the mice were housed in the pathogen-free facility of Bio-X Institutes at Shanghai Jiao Tong University. The experimental protocols and methods were ratified by the Animal Welfare Committee of the University [SYXK(SH)2011-0112]. All experimental methods were performed in accordance with the approved guidelines. Two and half- or six-month-old male mice were used for *in vivo* and *ex vivo* analysis. The compared mice were of the same age and same gender (male). Mice were anesthetized with Avertin (Sigma-Aldrich, St. Louis, USA) for blood sample collection. The animals were finally euthanized by cervical vertebra dislocation. Genotyping use the following sets of primers. *p38α*^*f/f*^ -F: 5′-TCCTACGAGCGTCGGCAAGGTG-3′; *p38α*^*f/f*^ -R: 5′-AGTCCCCGAGAGTTCCTGCCTC-3′; and LysM-Cre, oIMR3066: 5′-CCCAGAAATGCCAGATTACG-3′; oIMR3067: 5′-CTTGGGCTGCCAGAATTTCTC-3′; and oIMR3068: 5′-TTACAGTCGGCCAGGCTGAC-3′.

### Bone histomorphometry

Bone histomorphometry analysis was based on previously described protocol^56^. Firstly, calcein was intraperitoneally injected twice at 0.1 mg/10 g body weight at 1 and 8 days before the mice are sacrificed. Four percent paraformaldehyde (PFA) was used to fix the femurs overnight, which were stored in 70% alcohol for future use. The bones were dehydrated in 90–100% ethanol overnight in vacuum, and then transferred into xylene overnight. The femurs were embedded with resin, sliced into 4 μm sections, which were stained with Villanueva-goldner’s one step trichrome method. All bone-specific parameters were measured and expressed in units following the guidelines established by the American Society for Bone and Mineral Research histomorphometry nomenclature committee using OsteoMeasure software (OsteoMetrics Inc, Decatur, GA).

Fixed and ethanol-dehydrated femur were scanned with a high-resolution μCT (SkyScan1176 Software: Version 1.1 (build 6), Bruker, Kontich, Belgium). Data were acquired at 50 keV energy, 270 μA current, and 0.2 mm filter for 8.96 μm cubic resolutions. Three-dimensional reconstructions were generated with the following parameters: smoothing = 2, ring artifact correction = 8, minimum and maximum for CS to image conversion were 0 and 0.088, and threshold was 75 (spongiosa) or 115 (cortex). 3D structural parameters were obtained (1.5 mm) for trabecular (secondary spongiosa) and (0.5 mm) for cortical bone in femur. We measured Bone Mineral Density (BMD, mg/cm^3^), bone volume/trabecular volume (BV/TV, %), trabecular separation (Tb.Sp, μm), trabecular thickness (Tb.Th, μm), trabecular number (Tb.N, mm^−1^).

### Deoxypyridinoline Measurement

To evaluate bone resorption rate *in vivo*, urine Deoxypyridinoline (DPD) was determined with MicroVue (Quidel, San Diego, CA) assay kit following the protocol provided by the manufacturer. The final DPD values were normalized to the urine levels of creatine.

### Bone marrow colony forming unit assay

To determine colony forming units (CFUs), red blood cells were lysed and the washed bone marrow cells were plated at 5 × 10^6^ per well in 6-well plates. Three or four days later, complete αMEM medium with 10% FBS was changed and seven days later, the cells were fixed with 4% PFA for 30 min, and washed with PBS 3 times, then stained for ALP (Sigma-Aldrich, St. Louis, USA).

### *In vitro* osteoclastogenesis assay and bone resorption assay

Mouse bone marrow cells were flushed out from the limbs. The monocyte fraction was isolated by centrifugation on a Percoll plus lymphocyte separation medium gradient (Sigma-Aldrich, St. Louis, USA). The monocytes were counted and seeded into 96-well plate at 1 × 10^6^/well with 25 ng/ml M-CSF and 50 ng/ml RANKL in completed αMEM medium. Five to seven days later, the cells were fixed and stained for TRAP using the Acid Phosphatase, Leukocyte TRAP Kit (Sigma-Aldrich, St. Louis, USA). The cells were photographed and TRAP positive multinucleated osteoclasts were counted (≥3 nuclei).

Osteoclast resorption function was assessed by a pit formation assay on dentine slices (IDS, Boldon, UK). Monocytes were pre-cultured for 2 days in the presence of 25 ng/ml M-CSF and 50 ng/ml RANKL in completed αMEM medium, counted, and plated onto dentine slices that were preincubated with serum for 2 hrs. After 7 days, the dentine slices were sonicated in 0.5 M ammonium hydroxide, stained with Gill’s hematoxylin for 5 min, washed with water, and photographed under microscope. Then the resorbed areas were measured.

### MSCs and monocytes Co-culture

Co-culture experiments were done according to previously-described protocols^56^. Firstly, MSCs were counted and plated in 24-well plates at 5 × 10^4^ in the bottom. Twenty four hrs after cell adherence, monocytes or monocyte culture medium were added on top of the MSCs layer with or without RANKL/M-CSF in completed αMEM medium. Four to seven days later, the cells were fixed and stained for ALP.

### Cell Transfection

Monocytes were plated and transfected with siRNA for Creb (Santa Cruz, sc-35111, California), p38β (Santa Cruz, sc-39117, California), and control siRNA (Santa Cruz, sc-37007, California) using Lipofectamine 2000 (Invitrogen, USA). Cells were harvested and knockdown efficiency was determined with western blot analysis.

### Immunohistochemical staining and TUNEL assay

WT and p38α deficient monocytes were isolated and plated onto coverslips and were induced to enter differentiation process by M-CSF/RANKL. Cell proliferation and apoptosis were determined by Ki67 immunofluorescence staining (ab15580, Abcam, Cambridge, MA) and TUNEL assay (*In Situ* Cell Death Detection Kit Fluorescein, Roche, Basel, Switzerland) respectively.

### TRAP staining

To decalcify the tibia bones, the bones were immersed in 10% Ethylene diamine tetracetic acid (EDTA) at room temperature, which was changed every 2 days and continued for 3-4 weeks. After samples were soft, 70% to 100% alcohol was used to dehydrate the samples. Then the bone samples were dealcoholised in xylene for 5 hrs. The tibia bones were embedded in paraffin and cut into 5 μm sections. TRAP staining was carried out using Acid Phosphatase, Leukocyte TRAP Kit (387A, Sigma-Aldrich, St. Louis, USA) according to manufacturer’s protocol.

### Flow cytometry

The bone marrow monocytes were isolated from mutant and WT mice, with red blood cells lysed, and were analyzed using flow cytometry (FACSCalibur, Becton Dickinson, USA) to quantify the percentage of Gr1^low^CD11b^+^ monocytes.

### Cell proliferation assay

Cell number was assessed by CCK-8 assays. In brief, monocytes were plated at 5 × 10^4^/well into 96-well and cultured in the presence of 25 ng/ml M-CSF, up to 96 hrs. The CCK-8 was added to the cultures for 4 hours at 37 °C, and the cell numbers were determined by reading OD (optical density) at a wavelength of 450 nm using a microplate reader.

### Western blot analysis

TNEN buffer were used to lyse cells in the presence of protease and phosphatase inhibitors. SDS-PAGE was used to analyze protein lysates. The specific antibodies used in this study were: NF-κB p65 (Cell Signaling, 4767, Boston, MA), NF-κB p50/52 (Santa Cruz, sc-8414, California), p38α MAPK (Cell Signaling, 9212, Boston, MA), p38β (Cell Signaling, 2339, Boston, MA), p-p38MAPK (T180/182) (Cell Signaling, 9211, Boston, MA), p53 (c12) (Cell Signaling, 2524, Boston, MA), Tak1 (Cell Signaling, 4505, Boston, MA), p-Tak1(T184/187) (Cell Signaling, 4531, Boston, MA), Creb (Upstate, 05767, Boston, MA), p-Creb (Upstate, 6519, Boston, MA), ERK (Cell Signaling, 9107, Boston, MA), p-ERK (Cell Signaling, 9106s, Boston, MA), cFos (Calbiochem, pc38, Darmstadt, Germany), p-cFos (Santa Cruz, sc-81485, California), and β-ACTIN (Santa Cruz, sc-81178, California).

### Quantitative PCR

Cell or femur total RNA was extracted with Trizol reagent (Invitrogen, USA). cDNA was synthesized with Transcriptor First strand cDNA synthesis kit (Roche, Basel, Switzerland). Quantitative PCRs were carried out with FS Universal SYBR Green Master Premix (Roche, Basel, Switzerland). Endogenous GAPDH were used as an internal control. Quantitative PCR primers were listed in Supplementary Table S1.

### Chromatin immunoprecipitation (ChIP) assays

ChIP assays were carried out following the protocol from SimpleChIP™ Enzymatic Chromatin IP Kit (Cell Signaling, 9002, Boston, MA). Osteoclasts (4x10^7^) were treated with 1% formaldehyde for 10 min at room temperature to crosslink proteins to DNA, followed by the addition of glycine to stop the crosslinking. The cells were collected with a cell scraper and washed in ice-cold PBS. The chromatin was harvested and fragmented using enzymatic digestion. Briefly, resuspended cells were subjected to DNA shearing for 10 min at 37 °C. An aliquot of each sample was set aside as input control, while the remaining portion was subjected to immunoprecipitation with anti-Creb antibodies (Upstate, 05767, Boston, MA) overnight at 4 °C, with IgG (Beyotime, Jiangsu, China) as control. Immunoprecipitated complex was treated with protease and the DNA was amplified by PCR using primer pairs designed to amplify about 100 bp fragments spanning the 2 kb *BMP2* and *PDGF-AA* promoters. Aliquots were subjected to quantitative PCR using gene-specific primer sets that were listed in Supplementary Table S2.

### Data analysis

At least eight pairs of mice were used for bone histomorphometry analysis. For *ex vivo* assays, cells from 3 mutant mice were individually tested, with triplicate for each mouse. For cell line-based assays, experiments were repeated three times with triplicate for each. Total 9 readouts were obtained and statistically analyzed. Results are presented as mean ± SEM. Statistical comparisons were performed using unpaired Student’s two tailed t test. P < 0.05 was considered statistically significant. *p < 0.05, **p < 0.01 when mutant mice were compared to control mice, siRNA knockdown cells were compared to control cells.

## Additional Information

**How to cite this article:** Cong, Q. *et al*. p38α MAPK regulates proliferation and differentiation of osteoclast progenitors and bone remodeling in an aging-dependent manner. *Sci. Rep.* **7**, 45964; doi: 10.1038/srep45964 (2017).

**Publisher's note:** Springer Nature remains neutral with regard to jurisdictional claims in published maps and institutional affiliations.

## Supplementary Material

Supplementary Information
