# Supplementary material for: p38α MAPK regulates proliferation and differentiation of osteoclast progenitors and bone remodeling in an aging-dependent manner
Source: Sci Rep. 2017 Apr 6;7:45964. doi: 10.1038/srep45964 (PMC5382695; doi:10.1038/srep45964)
Supplement: Supplementary Information [file srep45964-s1.pdf]

Supplementary Information

p38α MAPK regulates proliferation and differentiation of osteoclast progenitors and bone remodeling in an aging-dependent manner

Qian Cong[1,2#], Hao Jia[3,2#], Ping Li[1,2], Shoutao Qiu[1,2], James Yeh[2], Yibin Wang[4], Zhen-Lin Zhang[2], Junping Ao[5], and Baojie Li[1,2*], Huijuan Liu[1,2*]

Supplementary Figure legends

Figure S1. p38α deficiency did not affect monocyte/osteoclast apoptosis or DC-Stamp expression.

A. TUNEL assays on p38α deficient and control monocyte/osteoclast cultures. Right panel: quantitation data. Scale bar, 50 μm. N=3.
B. Realtime PCR results showed that p38α deficient and control monocyte/osteoclast cultures showed similar levels of DC-Stamp. N=3.

Figure S2. SB203580 greatly inhibited osteoclast differentiation in p38α deficient and WT monocytes.

TRAP staining of SB203580-treated WT and p38α deficient monocyte cultures in the presence of M-CSF and RANKL (Upper panels). Bottom panel: Quantitation data. Scale bar, 200 μm. N=3. P-values are based on Student's t-test. **p<0.01 when the value of drugs-treated group compared to control group.

Figure S3. p38β knockdown did not significantly affect osteoclast differentiation.

A. Western blot analysis showed the protein levels of p38α and p38β in WT and p38α deficient monocyte cultures.
B TRAP staining of WT and p38α deficient monocyte cultures with p38β knocked down with siRNA, in the presence of M-CSF/RANKL (left panel). Upper right panel: western blot showing knockdown of p38β in WT and p38α deficient monocytes. Bottom right panel: quantitation data for TRAP positive osteoclasts. Scale bar, 200 μm. N=3.

Figure S4. LysM-Cre mediated p38α ablation did not affect body size and weight

A. Body weights and the lengths of limbs of 2.5-month-old LysM-Cre; $p38\alpha^{f/f}$ and control mice. N=8.
B. Body weights and the lengths of limbs of 6-month-old LysM-Cre; $p38\alpha^{f/f}$ and control mice. N=8.

Figure S5. Histomorphometry results revealed that LysM-Cre; p38α$^{f/f}$ mice showed distinct alterations in bone mass at 2.5 and 6 month of age.

A. A representative staining of femur bones of 2.5 month-old LysM-Cre; p38α$^{f/f}$ and control mice. Upper panel scale bar, 200μm. Bottom panel scale bar, 50 μm. N=8.
B. Two and half-month-old LysM-Cre; p38α$^{f/f}$ mice only showed a minor increase in BV/TV and a decrease in trabecular separation but not a change in trabecular number or thickness compared to control mice. N=8.
C. A representative staining of the femur bones of 6 month-old LysM-Cre; p38α$^{f/f}$ and control mice. Scale bar, 200 μm.
D. Six month-old LysM-Cre; p38α$^{f/f}$ mice showed a decrease in BV/TV, trabecular number, trabecular thickness, and an increase in trabecular separation compared to control mice. N=8

For all results in Fig. S5, P-values are based on Student's t-test. *p<0.05, **p<0.01 when the value of mutant mice or cells was compared to that of control mice or cells.

Figure S6. p38α deficient monocytes isolated from 6-month-oldmice behaved just like monocytes isolated from 2.5-month-oldmice.

A. p38α deficiency increased cell proliferation in monocyte/osteoclast cultures isolated from 6-month-oldmice. WT and p38α deficient monocytes were induced to differentiate by M-CSF/RANKL. At day 2, these cells were immunostained for Ki67 to detect S phase cells (left panel). The ratios of Ki67 positive cells to DAPI-stained cells were used as an indicator of cell proliferation rate. Right panel: quantitation data. Scale bar, 50 μm. N=3.
B. TRAP staining showed that p38α deficiency promoted osteoclast differentiation in low cell density culture but slightly inhibited osteoclast differentiation in high cell density cultures. Monocytes were isolated from 6-month-old LysM-Cre; p38α$^{f/f}$ and control mice, plated at different densities, and cultured in the presence of M-CSF/RANKL. After 7 days, the cultures were stained for TRAP (left panels). Right panels: quantitation data. Scale bar, 200 μm. N=3.

C. p38α deficiency did not affect resorbing activity of osteoclasts on dentine slices. WT and p38α deficient monocytes were isolated from 6-month-old mice and were induced to differentiate into osteoclasts by M-CSF and RANKL for 2 days. The osteoclasts were counted and the same numbers of cells were plated onto dentine slices. After 7 days, the dentine slices were sonicated and stained with Gill's hematoxylin. Bottom panel: quantitation data. Scale bar, 200 µm. N=3.

For all results in Fig. S6, P-values are based on Student's t-test. **p<0.01 when the value of mutant mice or cells was compared to that of control mice or cells.

Figure S7. Co-culture experiments showed that p38α deficient monocytes showed a decreased capacity in supporting MSC osteogenic differentiation.

A. WT MSCs were plated and 24 hrs later, monocytes isolated from 2.5-month-old WT and mutant mice were plated on top of the MSCs. These cells were co-cultured for 4 more days in αMEM in the absence of M-CSF/RANKL. The suspension cells were then washed off and the MSCs were stained for ALP. Bottom panel: quantitation of ALP. N=3.
B. WT MSCs were plated and 24 hrs later, monocytes isolated from 2.5-month-old WT and mutant mice were plated on top of the MSCs. These cells were co-cultured for 4 days in αMEM in the presence of M-CSF/RANKL. The suspension cells were then washed off and the MSCs were stained for ALP. Bottom panel: quantitation of ALP.

For all results in Fig. S7, P-values are based on Student's t-test. **p<0.01 when the value of mutant mice or cells was compared to that of control mice or cells.

Figure S8. p38α deficiency down-regulated TGFβ and IGF1 but not other coupling regulators tested in monocytes/osteoclasts.

A. p38α deficiency led to a decrease in the mRNA levels of TGFβ and IGF1 but failed to affect the mRNA levels of other coupling factors tested in p38α deficient monocytes/osteoclasts compared to control cells. N=3.
B. The mRNA levels of TGFβ were not altered during osteoclast differentiation

induced by M-CSF/RANKL.

C. The mRNA levels of IGF1 were not altered during osteoclast differentiation induced by M-CSF/RANKL.

For all results in Fig. S8, P-values are based on Student's t-test. **$p<0.01$ when the value of mutant cells was compared to that of control cells.

Supplementary Figure S1

A

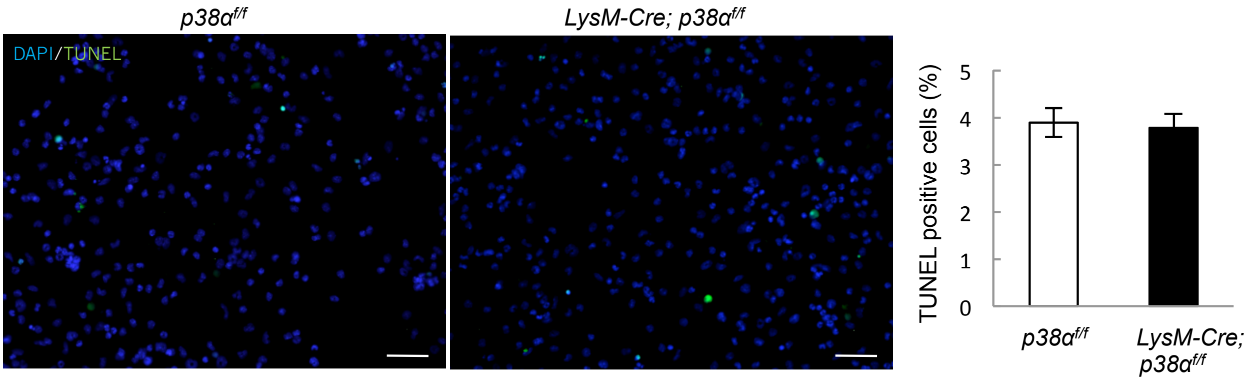

B

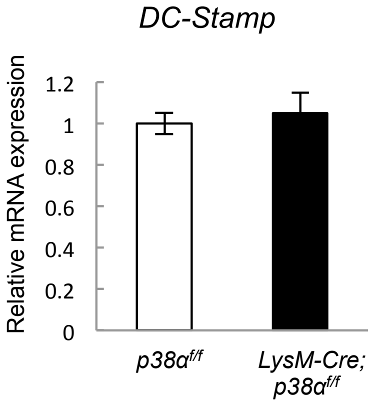

Supplementary Figure S2

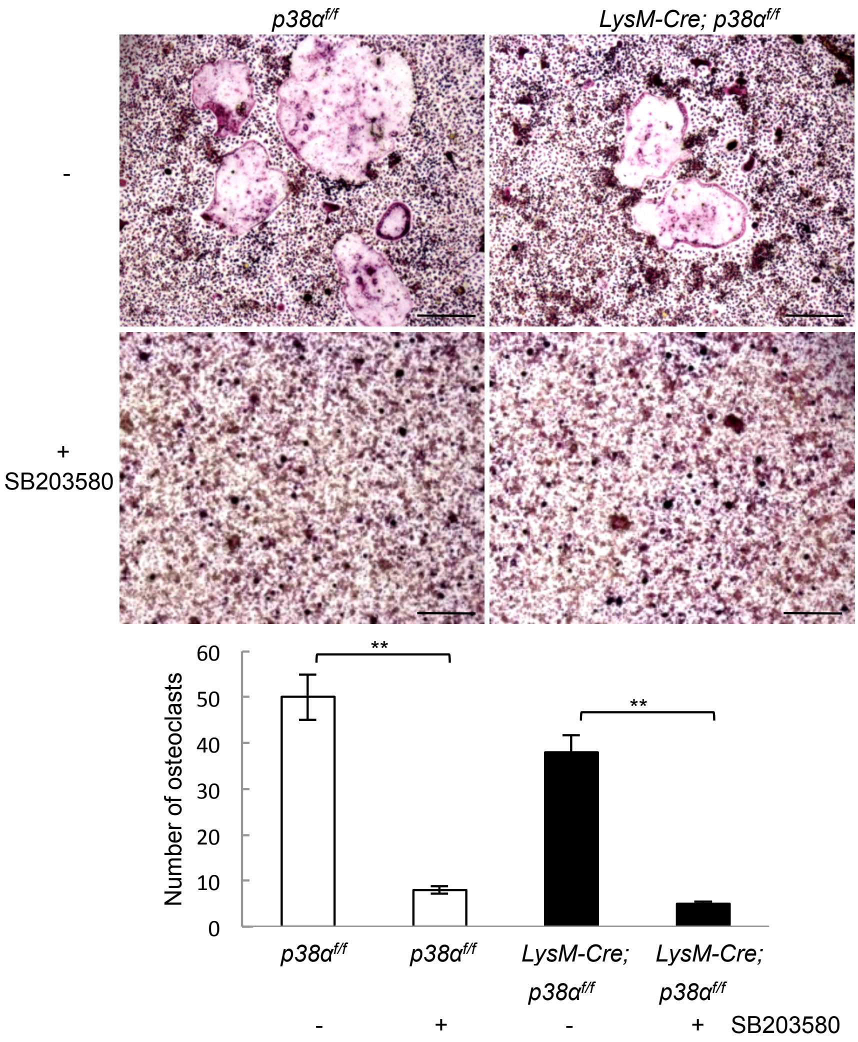

Supplementary Figure S3

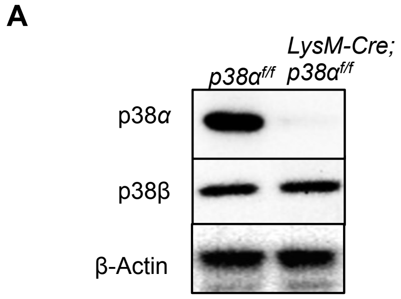

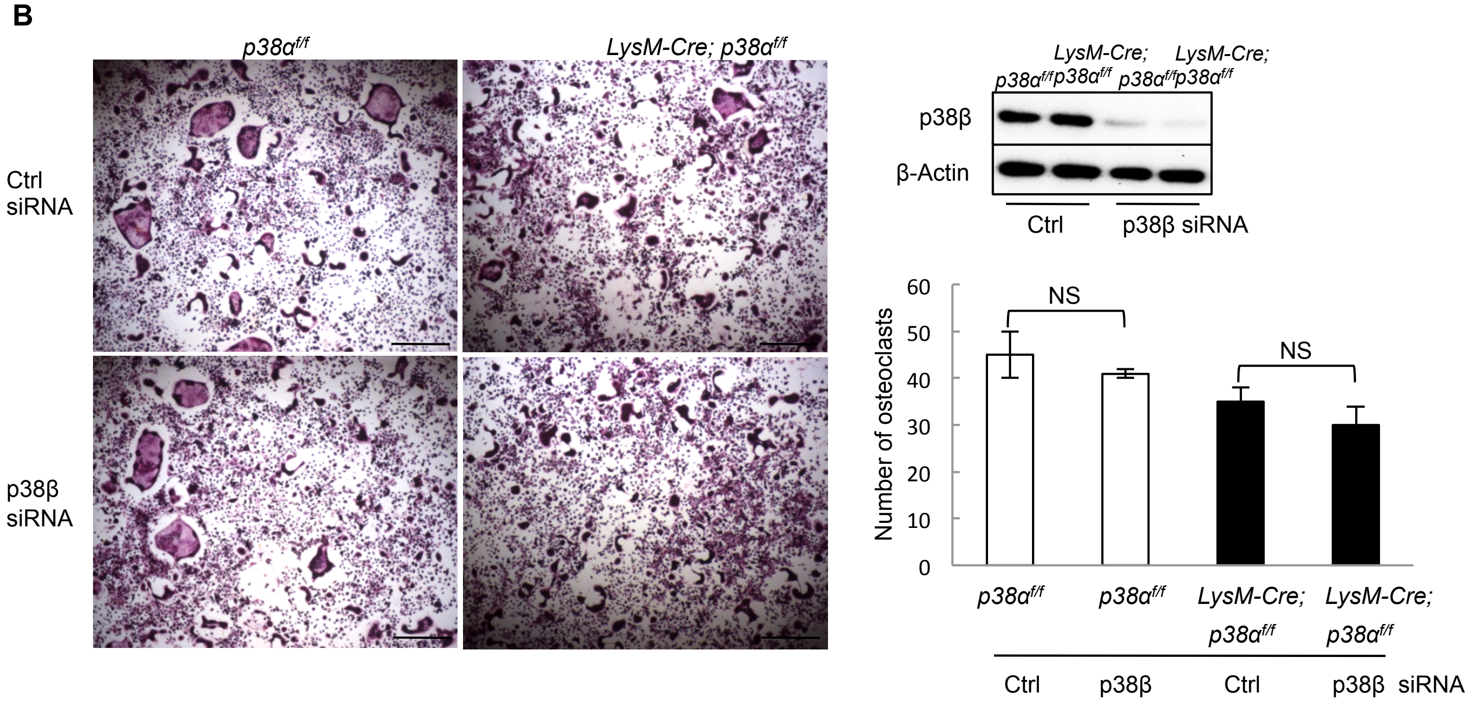

Supplementary Figure S4

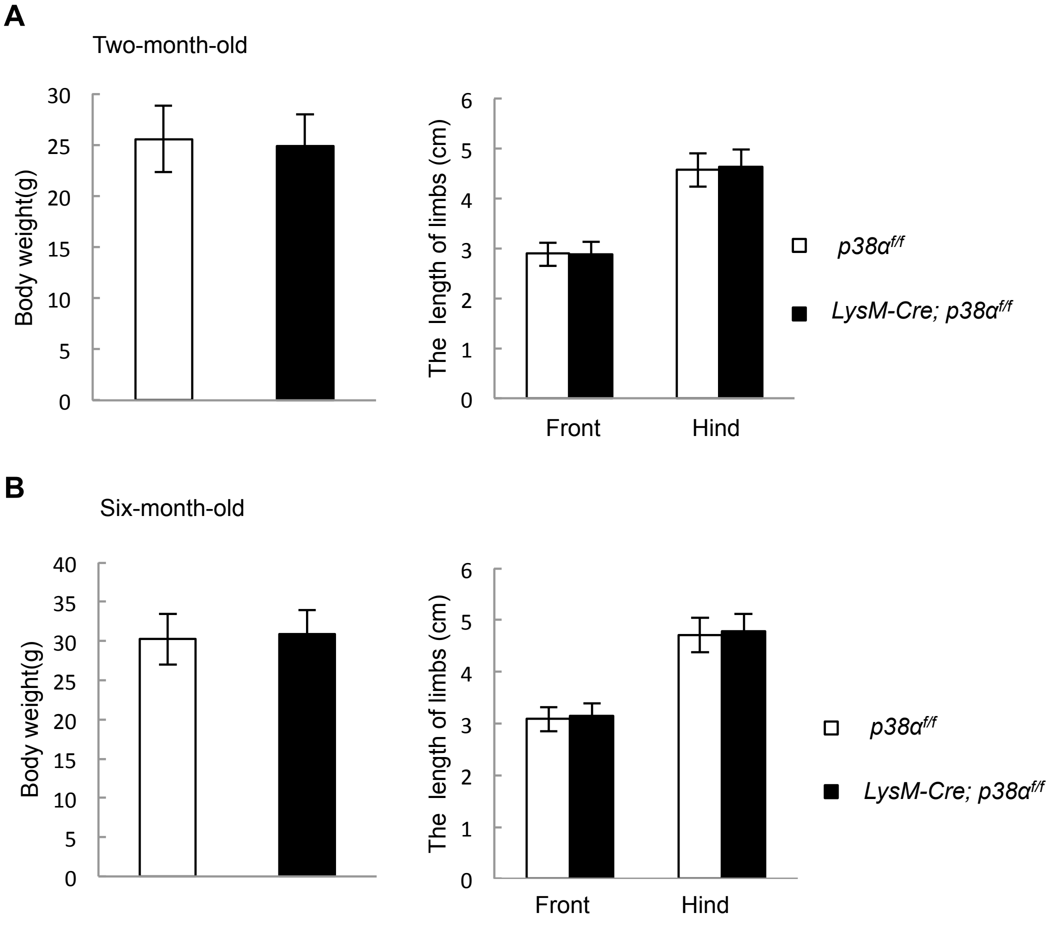

Supplementary Figure S5

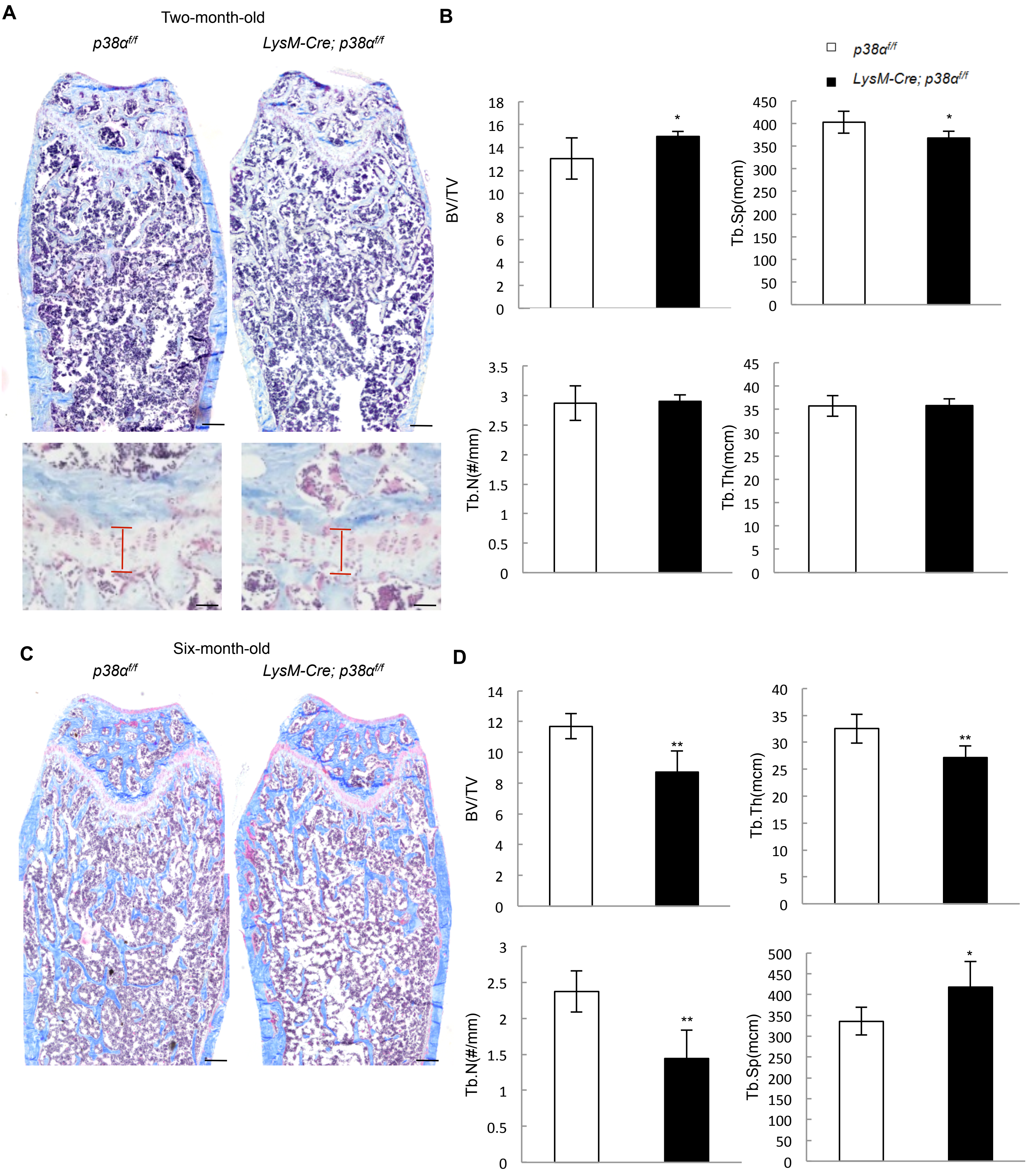

Supplementary Figure S6

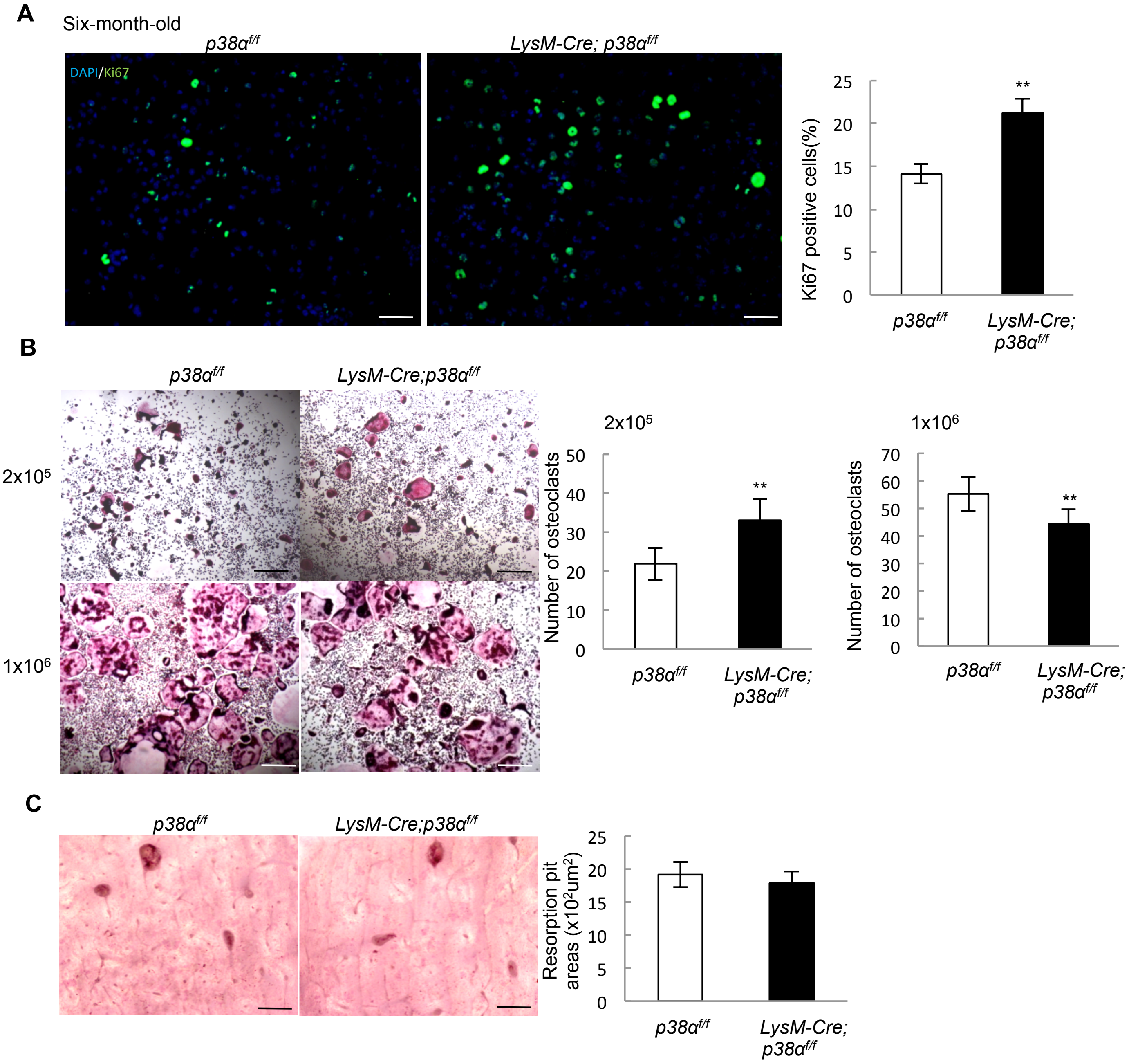

Supplementary Figure S7

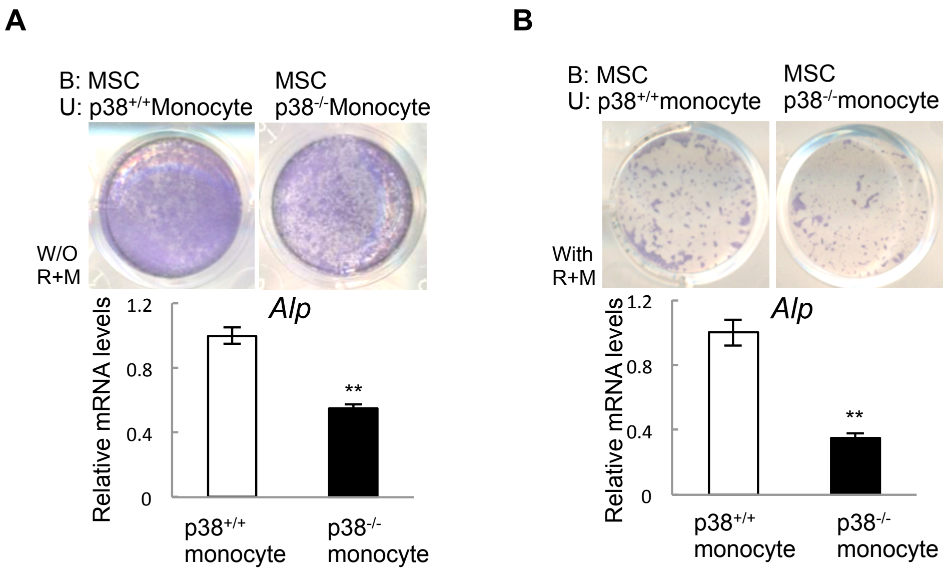

Supplementary Figure S8

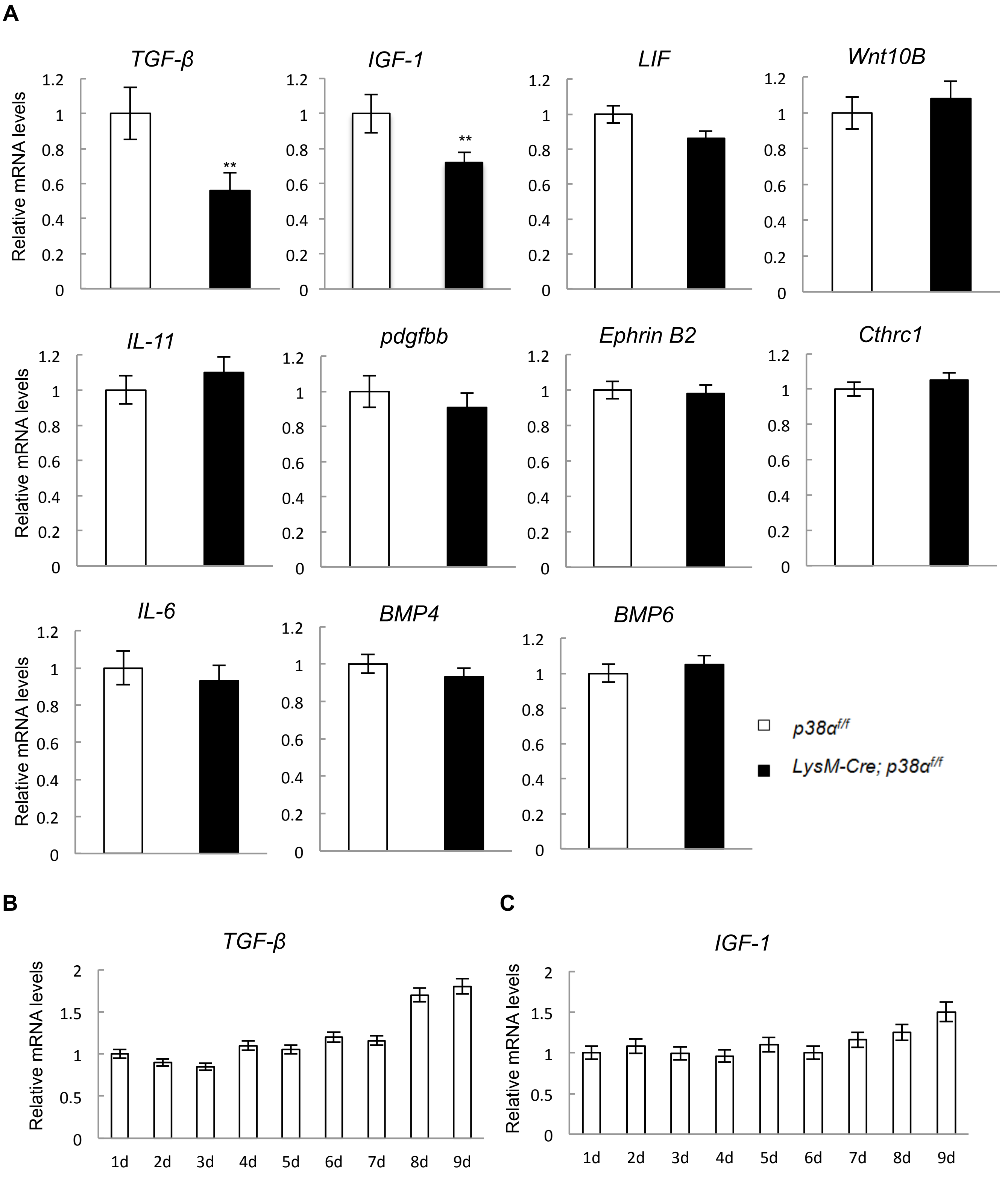

Supplementary Figure S9

Supplementary Figure S9A The full length blots for Fig. 1A

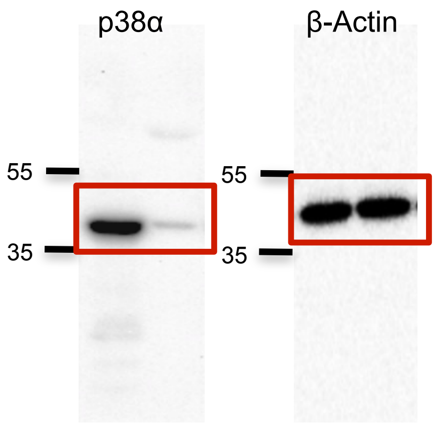

Supplementary Figure S9B The full length blots for Fig. 1D

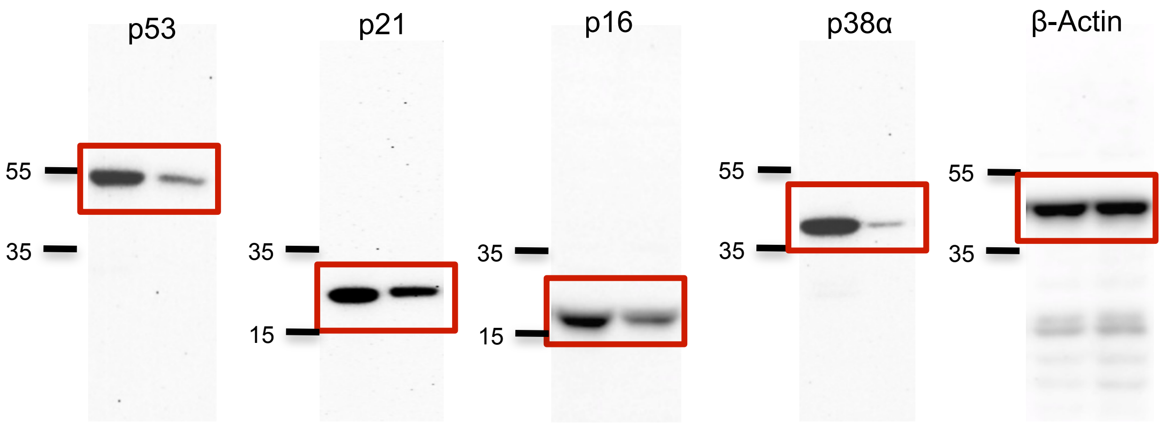

Supplementary Figure S9C The full length blots for Fig. 2D

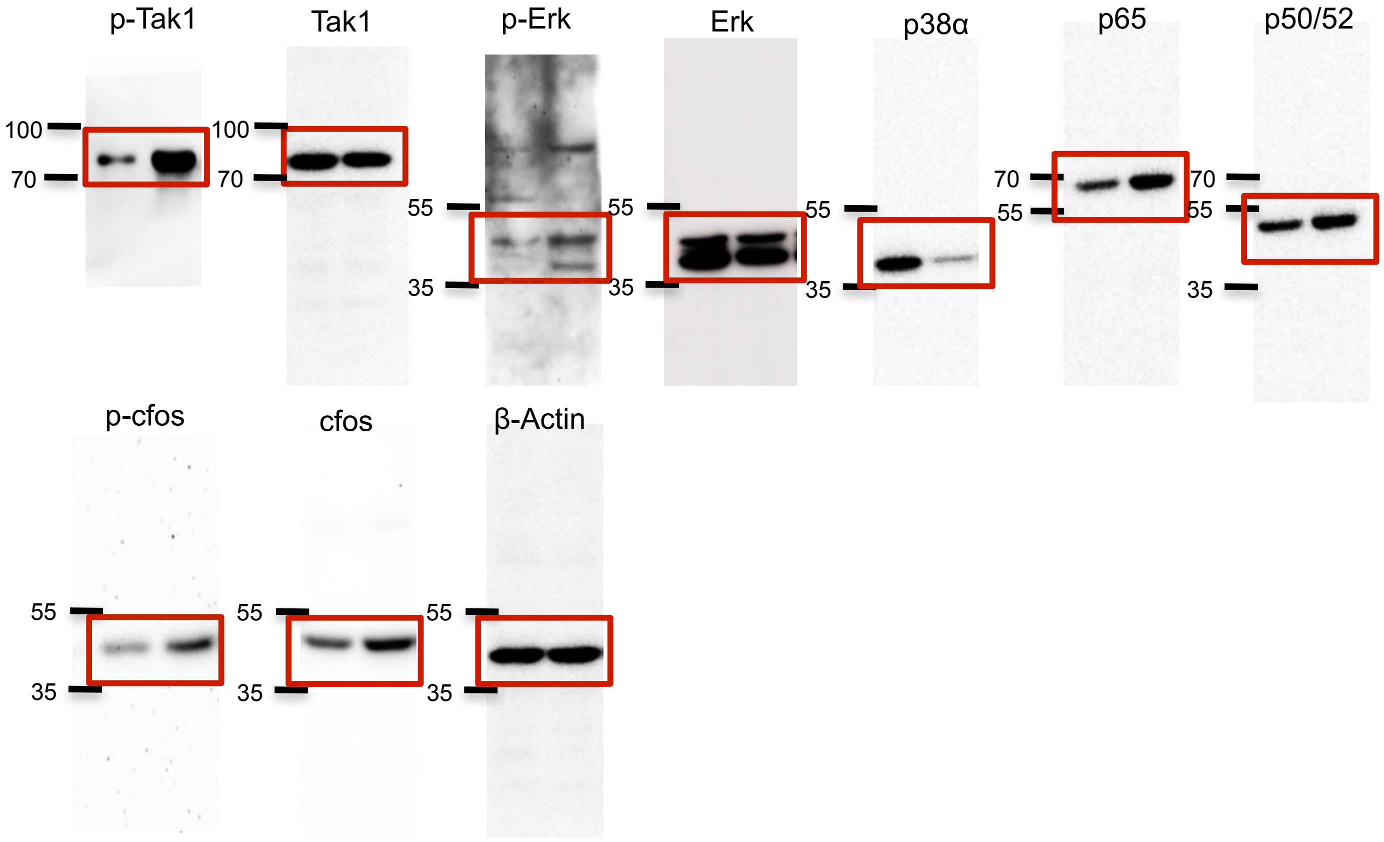

Supplementary Figure S9D The full length blots for Fig. 2E

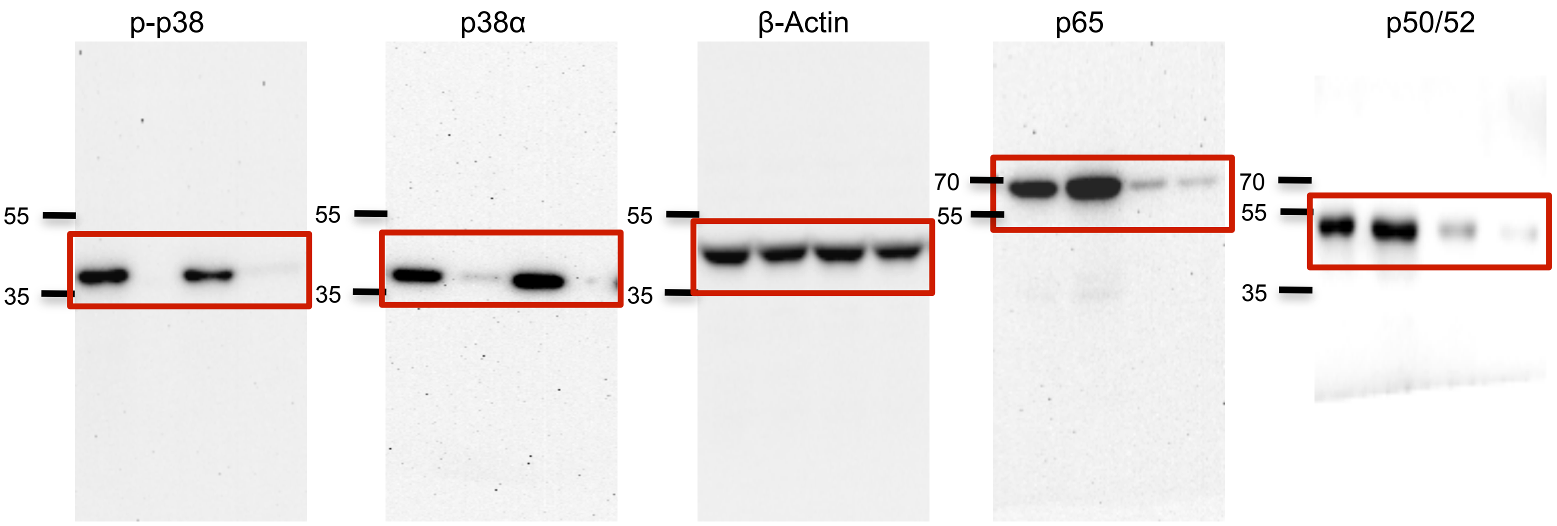

Supplementary Figure S9E The full length blots for Fig. 2F

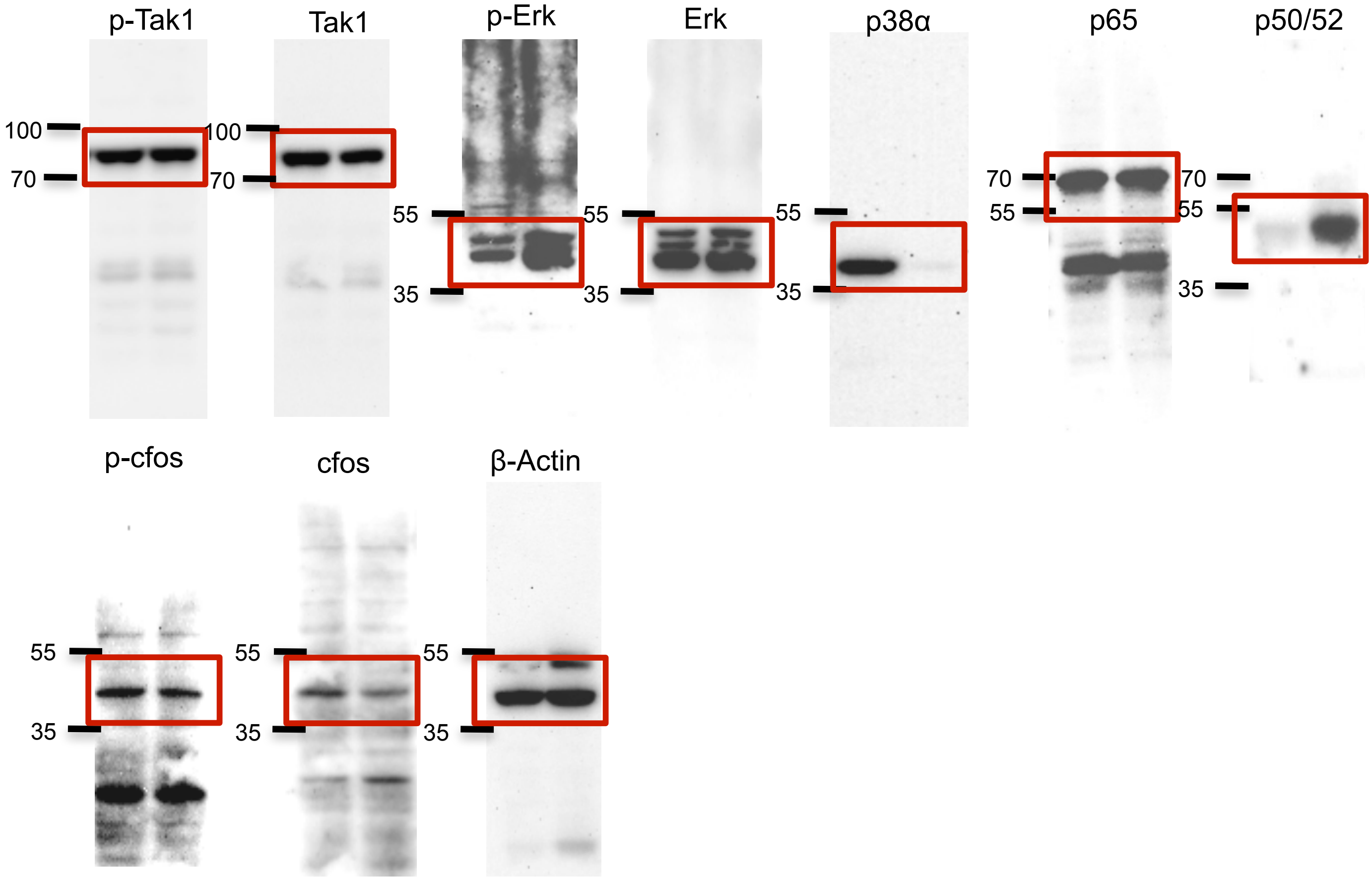

Supplementary Figure S9F The full length blots for Fig. 5C

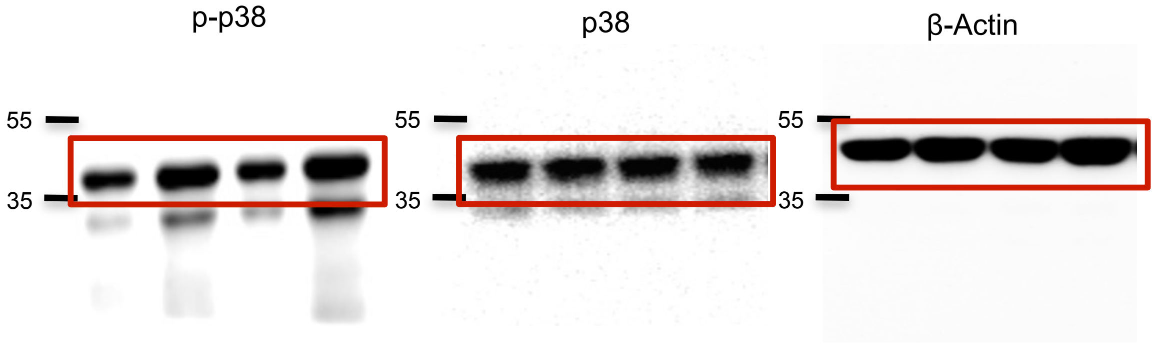

Supplementary Figure S9G The full length blots for Fig. 7F

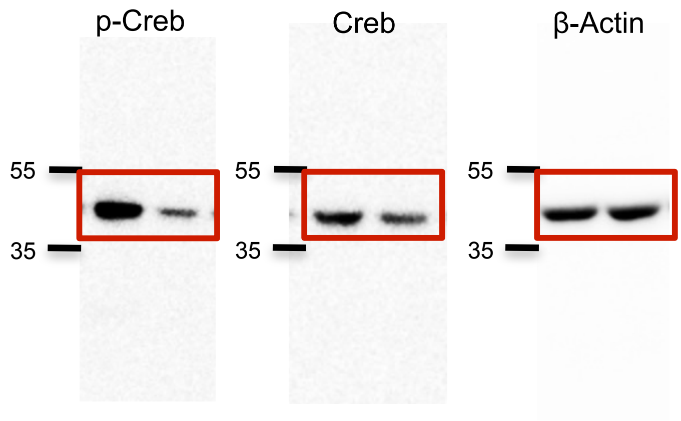

Supplementary Figure S9H The full length blots for Fig. 7G

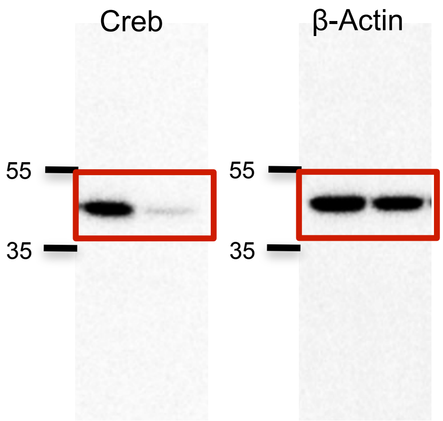

Supplementary Figure S9I The full length blots for Supplementary Fig. S3A

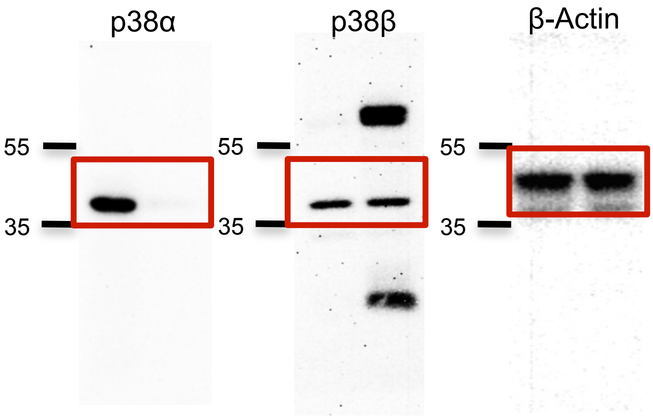

Supplementary Figure S9JThe full length blots for Supplementary Fig. S3B

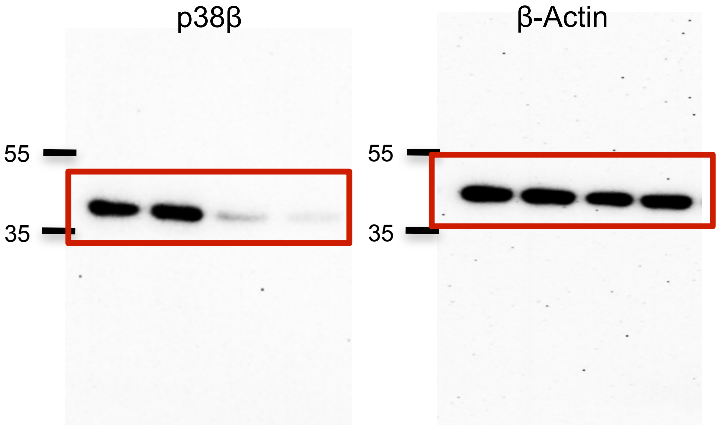

Supplementary Table 1 Quantitative PCR primers:

Primer	Forword 5'-3'	Reverse 5'-3'
Mitf	CCCTCTCACCTGTTGGAGTCA	CCGTTTCTTCTGCGCTCATAC
Pu.1	GATGGAGAAGCTGATGGCTTGG	TTCTTCACCTCGCCTGTCTTGC
c-Fos	CCTGCCCCTTCTCAACGAC	GCTCCACGTTGCTGATGCT
Nfatc1	CTCGAAAGACAGCACTGGAGCAT	CGGCTGCCTTCCGTCTCATAG
Trap	GCTGGAAACCATGATCACCT	GAGTTGCCACACAGCATCAC
Integrinβ3	CACCATCCACGACCGAAAAG	GGTACGTGATATTGGTGAAGGTAGAC
Pdgf-aa	GGATACCTCGCCCATGTTCTG	AATGACCGTCCTGGTCTTGCA
Pdgf-bb	GACCACTCCATCCGCTCCTTT	CTTGCACTCGGCGATTACAGC
IGF-1	TTTTCTATGGCAGCCTCAGTATTT	TCTCCCTCTTCTGGCAAAGTTAT
Bmp2	TGGAAGTGGCCCATTTAGAG	TGACGCTTTTCTCGTTTGTG
TGF-β1	AGCAACAATTCCTGGCGTTACCT	CGAAAGCCCTGTATTCCGTCTCC
IL-6	TTGCCTTCTTGGGACTGATGCT	GTATCTCTCTGAAGGACTCTGG
LIF	CAACCAACAACATGCGAGTG	GGTATTGCCGATCTGTCCTG
DC-Stamp	CTAAGGAGAAGAAAACCCTTG	CAGCATAGAAGACAACAATCC
Bmp4	CGAGAAGGCAGAGGAGGAG	CAAACTTGCTGGAAAGGCTC
Bmp6	ATGGCAGGACTGGATCATTGC	CCATCACAGTAGTTGGCAGCG
IL-11	GGACAGGGAAGGGTTAAAGG	GCTCAGCACGACCAGGAC
Wnt10B	GGCTGTAACCACGACATGGAC	ACGTTCCATGGCATTTGCAC
Ephrin B2	GACGTCCAGAACTAGAAGCTGG	CACCAGCGTGATGATGATGACG
Cthrc1	TGGACCAAGGAAGCCCTGAGT	TGAACAGGTGCCGACCCAGA
GAPDH	CCACAGTCCATGCCATCAC	CATACCAGGAAATGAGCTTGAC

Supplementary Table 2 Chromatin immunoprecipitation (ChIP) quantitative PCR primers:

BMP2 Primer	Forword 5'-3'	Reverse 5'-3'
0—-101site	aagtctctgtatttgtttcttc	taaatgaattctctgttctttg
-101—-201site	aactggattcacttctaggtcc	atttctctgaagagcactcgac
-201—-301site	tgggccaaacttgaggaaagtt	cagttactccaccttggtggtg
-301—-401site	tccagaggcatccattttacct	gccttgcagctttgggcctctg
-401—-501site	attgttgatgtcatcaccaaag	aatgagaaagttgcctttaatg
-501—-601site	taaatctcatataggttcggag	ttccttccctatcaagtttaat
-601—-701site	atggcttcagaggcgatcagcc	gtcactctgaatcatgagacac
-701—-801site	aacttctgcaacacgttttaaa	aggaaggctgtctacaagggaa
-801—-901site	tcaaaaataccttatttgacct	aactctaggttcgttcacctaa
-901—-1001site	attttagctccccgactgaaaa	ctttccagtttgcaaagcaaag
-1001—-1101site	atctggttcacaacgtaacgtt	ttcatagtaccaccactggcta
-1101—-1201site	ggctcctgaatgtagggagaaa	tggccaagccagttttcttcct
-1201—-1301site	cagccagagccttactgctgga	actccagagtcaggtaacaaga
-1301—-1401site	aggtgggtgtggaagggaagat	tgtgtccatgtgaggggacaat
-1401—-1501site	cacacacacacacacacacaca	attataaaaatacgggtacagg
-1501—-1601site	ttacataaataaatacatata	tgagtgcattattacattttat
-1601—-1701site	gatgtgtatcggctatttctcg	acagaacttacaagttattaga
-1701—-1801site	tcttctttttaatgtgctctta	ctagggcaaatccagagtccac
-1801—-1901site	tctcccgcagctgtgggcgcgg	gagcggggcggggcgagcgcgc
-1901—-2001site	caccgcggccgccccgtagggc	aacacctccccctcggaggcgc

PDGF-AA Primer	Forword 5'-3'	Reverse 5'-3'
0—-101site	tcccgaggtgttagggctgggg	gtggggggaggggggtagctgaa
-101—-201site	gcccaccaggtatttggagcta	ggagcctttcatccccagcttc
-201—-301site	tgcccggctgcgcctttgtctg	gtccagttcactccctttcatg
-301—-401site	aggctgtatccagttgcatccc	aaaggtccacaatcctctaacc
-401—-501site	cgagttaaggagctgacaggac	gaagtctgatcagggctggacg
-501—-601site	tgtccgcatgtgccttccccgc	aggaactcctgggttctacctt
-601—-701site	caccagccgcttcttttctatc	ctgcaccctccagatccttagc
-701—-801site	ggtgtcaaggtggcgatgaggc	tgggacacctgattgaggaact
-801—-901site	ggctttggctctggggcgcggg	aggggagtgagtctttgcgga
-901—-1001site	ccttttatggagaggggaaggc	gccaaaaagggcatgagagtcg
-1001—-1101site	gcagcagctctgggcgctgccc	ggcggggaggggaggaggccgc
-1101—-1201site	cggtgccgcaggattgcagctg	cggcgcccgcggctcggagccc
-1201—-1301site	agcttggatggatgtagctgcc	gaagcgctcaggggtctcgcgg
-1301—-1401site	cgaggtgcgggtcccgggcccg	cggccggcgacagggagggggt
-1401—-1501site	gctccacacgcgcgtcctgcgg	gcagcagccgcgggaagaccag
-1501—-1601site	tgcggcggcagcggcgccagct	cgcgccgcctggcagcagccta
-1601—-1701site	gccgcgggagctgcccggcccc	ggcgcggcccggcggcctgcgt
-1701—-1801site	gtgctgggccgcgccgtgcccg	cctcgcctctccctgagggctc
-1801—-1901site	cggtagctggtaccgggccgag	ttgctgccgtcagccccgggag
-1901—-2001sit	atatgcggatttaccggccggg	gggctgggcttcttccttggtg
